# Paracetamol (acetaminophen) use in infants and children was never shown to be safe for neurodevelopment: a systematic review with citation tracking

**DOI:** 10.1007/s00431-022-04407-w

**Published:** 2022-02-17

**Authors:** Jasmine Cendejas-Hernandez, Joshua T. Sarafian, Victoria G. Lawton, Antara Palkar, Lauren G. Anderson, Vincent Larivière, William Parker

**Affiliations:** 1 Department of Surgery, Duke University School of Medicine, Durham, NC USA; 2 WPLab, Inc, 1023 Wells St, Durham, NC 27707 USA; 3 École de Bibliothéconomie Et Des Sciences de L’information, Université de Montréal, Montreal, Canada; 4 Duke Global Health Institute, Duke University and Duke University Medical Center, Durham, NC 27710 USA

**Keywords:** Behavior, Neurodevelopment, Infant, Child, Autism

## Abstract

Although widely believed by pediatricians and parents to be safe for use in infants and children when used as directed, increasing evidence indicates that early life exposure to paracetamol (acetaminophen) may cause long-term neurodevelopmental problems. Furthermore, recent studies in animal models demonstrate that cognitive development is exquisitely sensitive to paracetamol exposure during early development. In this study, evidence for the claim that paracetamol is safe was evaluated using a systematic literature search. Publications on PubMed between 1974 and 2017 that contained the keywords “infant” and either “paracetamol” or “acetaminophen” were considered. Of those initial 3096 papers, 218 were identified that made claims that paracetamol was safe for use with infants or children. From these 218, a total of 103 papers were identified as sources of authority for the safety claim.

*   Conclusion*: A total of 52 papers contained actual experiments designed to test safety, and had a median follow-up time of 48 h. None monitored neurodevelopment. Furthermore, no trial considered total exposure to drug since birth, eliminating the possibility that the effects of drug exposure on long-term neurodevelopment could be accurately assessed. On the other hand, abundant and sufficient evidence was found to conclude that paracetamol does not induce acute liver damage in babies or children when used as directed.

**Table:** 

**What is Known:** *• Paracetamol (acetaminophen) is widely thought by pediatricians and parents to be safe when used as directed in the pediatric population, and is the most widely used drug in that population, with more than 90% of children exposed to the drug in some reports.* *• Paracetamol is known to cause liver damage in adults under conditions of oxidative stress or when used in excess, but increasing evidence from studies in humans and in laboratory animals indicates that the target organ for paracetamol toxicity during early development is the brain, not the liver.*	
**What is New:** *• This study finds hundreds of published reports in the medical literature asserting that paracetamol is safe when used as directed, providing a foundation for the widespread belief that the drug is safe.* *• This study shows that paracetamol was proven to be safe by approximately 50 short-term studies demonstrating the drug’s safety for the pediatric liver, but the drug was never shown to be safe for neurodevelopment.*

**Graphical abstract:**

Paracetamol is widely believed to be safe for infants and children when used as directed, despite mounting evidence in humans and in laboratory animals indicating that the drug is not safe for neurodevelopment. An exhaustive search of published work cited for safe use of paracetamol in the pediatric population revealed 52 experimental studies pointing toward safety, but the median follow-up time was only 48 h, and neurodevelopment was never assessed.

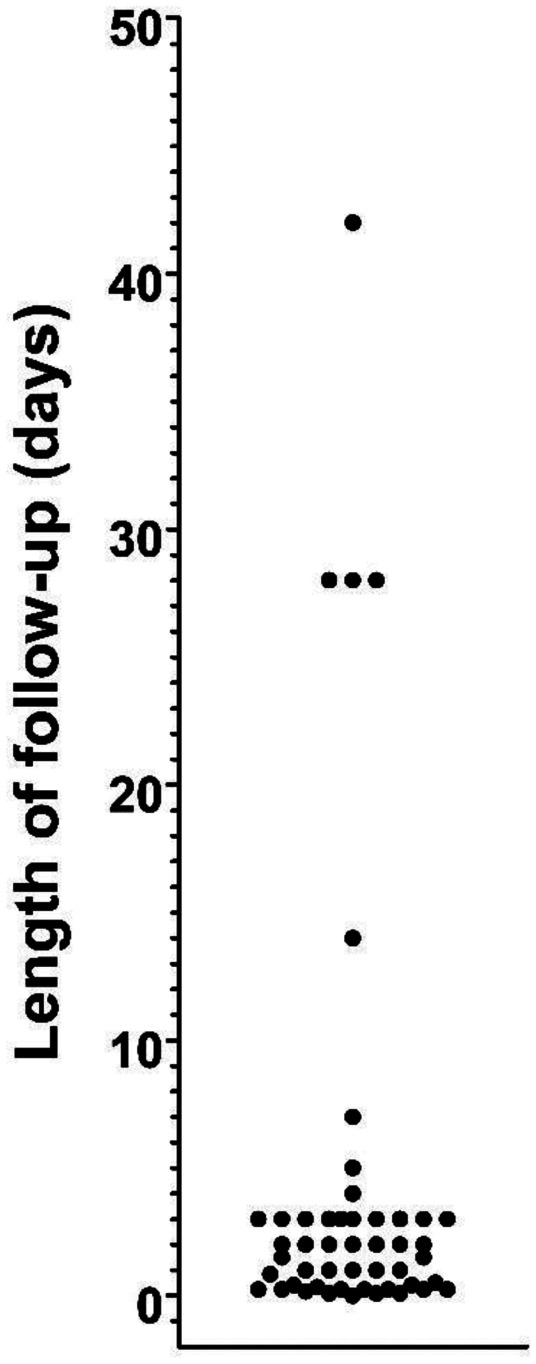

**Supplementary information:**

The online version contains supplementary material available at 10.1007/s00431-022-04407-w.

## Background

Most parents and pediatricians currently believe that use of paracetamol in infants and children is safe, promulgating widespread use of the drug in that population. Use of paracetamol in the pediatric populations now exceeds 90% in some studies [1] and persists even under circumstances in which the drug may have no benefits, such as prophylaxis prior to some vaccinations [2] and treatment of mild fevers [3]. Such beliefs and practices are strengthened and supported by a medical literature which repeatedly asserts without reservation that, when used as directed, the drug is safe in the pediatric population. However, mounting evidence points toward the view that paracetamol exposure during early development can have an adverse effect on neurodevelopment, even when used as directed. For example, in a recent review [4], eight studies supporting a link between prenatal paracetamol exposure and neurodevelopmental problems were identified [5–12]. In the 3 years since that review, at least six additional studies have confirmed this same relationship, three of which have used data from the Norwegian Mother and Child Cohort Study [13–18]. Although exposure to paracetamol in utero is associated with neurodevelopmental problems, even after consideration of potentially confounding factors, the effects are typically small, and the amount of paracetamol required to yield the effect is greater than the amount typically used by average individuals. For example, after adjusting for potential confounders such as parental education level, use of vitamin supplements, parental BMI, smoking, and use of other drugs, Skovlund and colleagues found a weak yet significant association between prenatal exposure to paracetamol and mother-reported communication skills: the chances of being in a lower development category increased with increasing periods of prenatal paracetamol use but not prenatal opioid use [13]. In another example, using propensity score matching, Vlenterie and colleagues found that 28 or more days of paracetamol use during pregnancy was associated with a modestly increased risk of delayed motor milestone attainment (OR: 1.35, 95% CI 1.07–1.70) by children at 18 months [14]. 

Evidence points toward a higher risk of paracetamol-induced neurodevelopmental disorders when exposure occurs after birth as compared to in utero. Studies using laboratory rodents demonstrate that exposure to near therapeutic doses of paracetamol during the first days of life induces profound, long-term neurological changes [19, 20], whereas somewhat higher doses are required to induce permanent neurological damage during pregnancy [21]. These laboratory studies demonstrate that the target organ for toxicity in neonates is the central nervous system, not the liver, and demonstrate that if paracetamol had been tested using current guidelines, it would never have been approved for use in children. More concerning are observations in children indicating that paracetamol is not safe for neurodevelopment. The 2008 study which first raised a red flag regarding the safety of paracetamol during neurodevelopment found a greater than 20-fold risk of regressive autism with paracetamol use during childhood [22]. Although this relatively small study did not attract enough interest to promote larger studies, other lines of evidence support the view that paracetamol exposure during early life can lead to neurodevelopmental disorders. For example, a startling twofold greater incidence of infantile autism in circumcised boys compared to non-circumcised boys [23] can be readily explained by potentially negative impacts of paracetamol exposure during and following the circumcision procedure [4]. Sadly, the widely held and entrenched belief that vaccines induce autism [24, 25] may be yet another result of the impact of paracetamol on neurodevelopment in combination with widespread use of the drug during vaccination [4].

With the above concerns in mind, a systematic evaluation of the peer-reviewed literature was initiated to address the question of why paracetamol is widely believed to be safe for use during early development. All papers published between 1974 and 2017 that contained the keywords “infant” and either “paracetamol” or “acetaminophen” were considered. All papers which made claims that paracetamol or acetaminophen is safe for use in infants or children were identified, and the justification for this claim was critically evaluated.

The use of paracetamol predates current safety standards used in the pharmaceutical industry, and even if current standards were applied, those standards do not mandate testing for long-term neurological development. Thus, to those aware of the inner workings of the drug approval process, especially as it has been applied to paracetamol, the results obtained in this study will not be surprising and may even be considered by some to be a foregone conclusion that need not be evaluated. Nevertheless, the widespread belief that paracetamol is safe and the resulting widespread use of the drug in the pediatric population is built on the assumption that it is safe for neurodevelopment. With this in mind, this review focuses on unqualified claims of safety in the medical literature that, reasonably, are taken by parents and even many physicians to indicate that the drug is safe for neurodevelopment.

## Methods

As a first step in understanding why paracetamol is thought to be safe during early development, all titles and abstracts in the PubMed^®^ Database with keywords “infant” and “acetaminophen or paracetamol” published between 1974 and 2017 were identified. The term “infant” rather than “child” was selected because (a) the number of papers with the term “child” was prohibitively large, and (b) the focus of the study was intended to be on drug exposure during early development, from birth to age approximately 6 years, not individuals up to the age of 17 years. In all cases, the terms paracetamol and acetaminophen were taken to be synonymous, and no distinctions were made.

In the second step, two coauthors (JCH and JTS) independently screened all titles and abstracts. In this step, articles that could not be obtained in English and all articles not describing use of paracetamol in humans were eliminated from the study. Based on titles and abstracts (if available), articles were tagged which were deemed likely to make claims regarding the safety of paracetamol use in infants and children between birth and age 6 years.

In the third step, two coauthors (JCH and JTS), continuing to work independently, examined full texts of all tagged titles and abstracts. Texts were examined for the following three assertions:

- Paracetamol use is “safe” in children or infants.
- Paracetamol is the “drug of choice” in children or infants.
- Paracetamol use is “recommended” for children or infants.

In cases where the terms “drug of choice” or “recommended” were used, the context was considered. In some cases, particularly in manuscripts expressing caution regarding the use of paracetamol, these terms were not taken to imply safety, but rather were taken to be an indicator of the common acceptance of the drug. These articles were excluded from the study. Based on this approach, articles were tagged that were considered to have made safely claims regarding the use of paracetamol in infants or children younger than 6 years old.

**Fig. 1:**
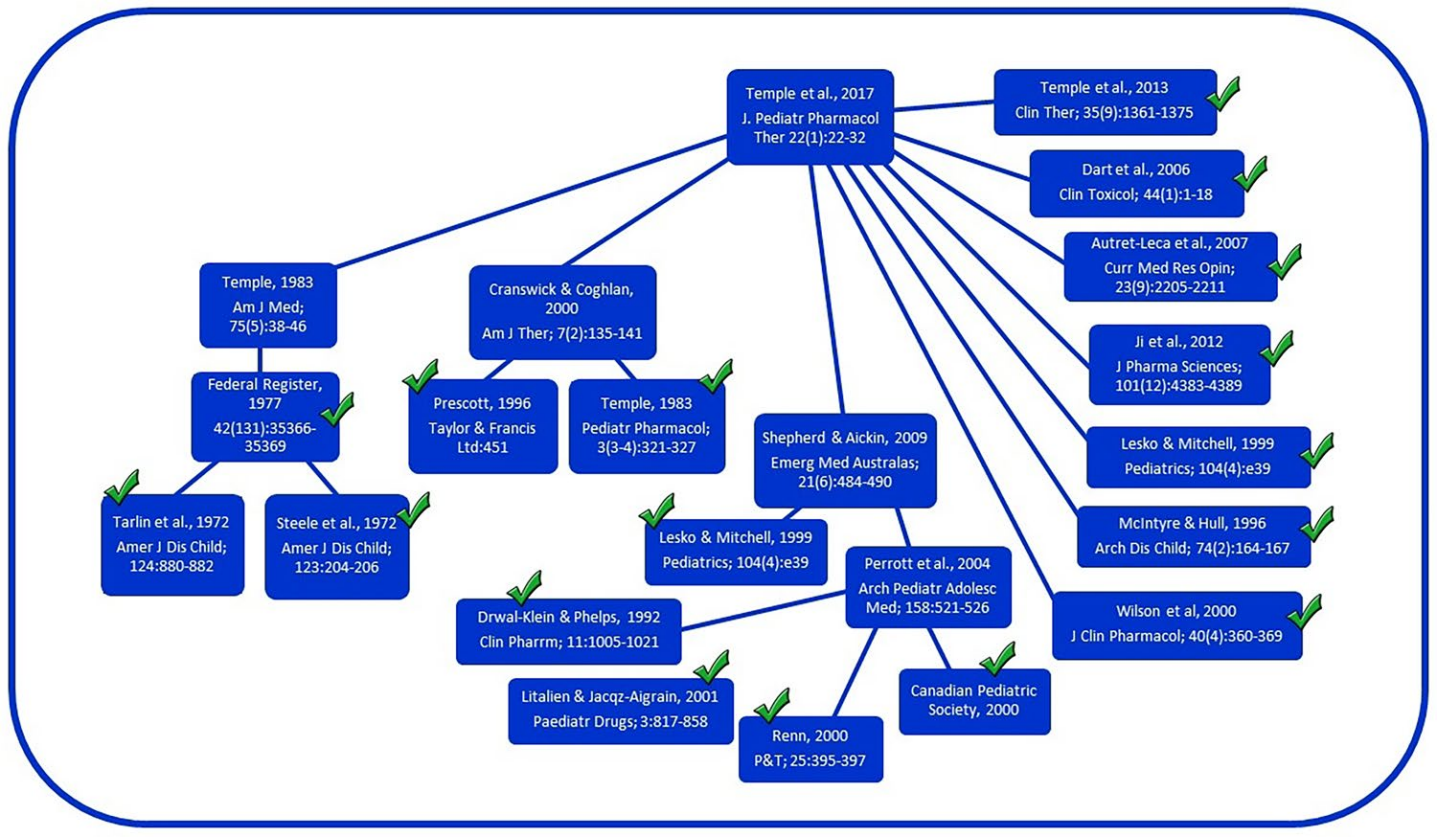
Flow diagram illustrating connections between articles claiming that paracetamol use is safe for infants or children when used as directed. In this example, the citations in a paper by Temple and colleagues in 2017 [30] are assessed. Articles describing new experiments designed to test safety of paracetamol or which contain claims of safety without citation are included in Table 2 and are indicated by a check mark. Articles shown in the diagram which do not describe experiments designed to test safety of paracetamol and which cite other articles as a source for claims of safety [27, 31, 32, 159] are not included in Table 2 and are not indicted by a check in the diagram

Still working independently, two coauthors (JCH and JTS) evaluated each manuscript making a claim of safety, determining the source of authority for the stated claim. If no literature was cited to support the claim, this was documented. In cases where the source that was cited contained another citation, that secondary reference was obtained and evaluated. This process continued as needed until an original source or sources describing an actual demonstration of safety was identified. An example of the results of this process is shown in Fig. 1.

In the fourth step, any discrepancies between the analyses provided by coauthors JCH and JTS were arbitrated by coauthor WP. In the fifth step, articles upon which safety claims were based were compiled. Finally, articles which made safety claims and articles upon which safety claims were based were evaluated for actual experiments designed to assess safety. For each experiment described, the study group, endpoints measured, and follow-up time were evaluated. Data were graphed and descriptive statistics calculated using GraphPad Prism 8 software. The review was not registered, and the protocol is as described in this Methods section.

## Results

An overview of results from a systematic search for studies demonstrating safety of paracetamol use in infants and children is shown in Table 1. The initial Medline search provided 3096 articles that contained the terms infant and either paracetamol or acetaminophen that were published between 1974 and 2017. From these articles, 467 were selected for assessment based on likelihood of safety claims regarding use of paracetamol in infants or children. Of these 467 articles, 218 made safety claims regarding the use of paracetamol in infants or children. During this phase of the study, numerous articles were identified which either claimed or demonstrated that paracetamol use, even at doses beyond the recommended dose, does not generally cause long-term liver damage in infants or children. Any claims of safety for liver function were not evaluated in detail and were not considered in this study. Only general claims of safety were assessed.

**Table 1:** Number of citations identified in the systematic search during each step of the study. Numbers are provided for both analysts performing the work (JCH and JTS). The overlap is the number of citations that were the same between the two analysts

Step	JCH	Overlap	JTS	Total
1. Medline (paracetamol + infant)				3096
2. Safety claim, first step	310	193	350	467
3. Safety claim, second step	189	144 (53*)	189	234
4. Safety claim, final	173	128 (37*)	173	218
5. Sources attributed to safety claim				103
6A. Sources with experiments supporting safety claim				36
6B. Safety claim, not cited as a source, with experiments supporting safety claim				16

*Numbers in parentheses indicated the number of citations in which both analysts identified the same citation, but not the same source or sources as the authority for claims of safety.

Of the 218 articles making claims that paracetamol use in infants or children is safe, half (114) provided no citation. The other half (114) of the articles cited additional articles as evidence that paracetamol is safe in infants or children. Articles making safety claims as well as articles cited as sources of authority for safety claims were evaluated as described in the Methods. In some cases, the “primary” cited articles did not make original claims of safety, but rather cited additional (“secondary”) articles. In cases where a primary article cited another article, the primary article was not considered to have made an original claim of safety, and was not evaluated further. An example of the results of this process is shown in Fig. 1. Both primary articles and secondary (and tertiary, etc.) articles attributed with claims of the safety of paracetamol use in infants or children were compiled and are shown in Table 2. In total, 103 articles were identified which were cited as containing original claims that paracetamol use in infants or children is safe when used as directed. In addition, 16 of the 218 articles with safety claims (a) made those claims based on original experimental evidence and (b) were not cited by other papers. These articles are also included in Table 2, listed at the bottom of the table with zero citations.

**Table 2:** Sources of authority for the assertion that paracetamol is safe for infants or children when used as directed

**Sources cited for safety of paracetamol in children or infants: study number, year, and design**	**Study subjects**	**Outcome measures related to safety or safety claims made**	**Duration of monitoring**	**Times cited**
1. 1999: Double-blind clinical trial with three treatments, one of paracetamol and two different concentrations of ibuprofen given for fever [26]	9127 children treated with paracetamol; median age is 14 months	“Serious adverse clinical events” requiring hospitalization: gastrointestinal bleeding, renal failure, anaphylaxis, Reye’s syndrome, asthma, bronchiolitis, and vomiting/gastritis	4 weeks	13
2. 1997: Randomized, double-blind, three-way crossover study with three treatments, one each of paracetamol, ibuprofen, and placebo given by parents for headache. Each child with three migraine attacks was treated in random order with single oral doses of the study drugs [44]	80 children were treated with paracetamol, age range approximately 4 to 16 years	Monitoring by parents for “adverse events”: nausea, vomiting, and gastric pain	2 h: all patients received paracetamol at some point, so long-term monitoring was not feasible	9
3. 1978: Editorial describing current practice with analgesic use in children [45]	NA: Editorial	Claims: “anticipated liver damage is not observed” based on personal experience and interactions with other clinicians	NA: Editorial	8
4. 2001: Review describing analgesic use in children [46]	NA: Review	Claims: “40-year safety record in children” without citation	NA: Review	8
5. 1978: Review describing antipyretic therapy in febrile children [47]	NA: Review	Claims: “relatively free of adverse reactions” without citation and 9 citations provided for the statement that hepatotoxicity from paracetamol in children is “very low compared with that seen in adults.”	NA: Review	7
6. 2000: Renn, 2000 Erroneous citation*	NA: Erroneous citation	NA: Erroneous citation	NA: Erroneous citation	7
7. 2011: Report describing current recommended practice [48]	NA: Report	Claims: “generally regarded as safe” without citation. Lesko (1999) is cited for equivalent safety between ibuprofen and paracetamol	NA: Report	7
8. 1983: Review describing pediatric dosing of paracetamol [49]	NA: Review	Claims: “one of the safest” without citation	NA: Review	6
9. 1995: Double-blind clinical trial with three treatments, one of paracetamol and two of different concentrations ibuprofen given for fever [50]	28,130 children treated with paracetamol; median age is 40 months	Serious events defined as hospitalization for acute gastrointestinal bleeding, acute renal failure, or anaphylaxis	4 weeks	6
10. 1998: Practice guidelines** [51]	NA: Practice guidelines	Claims: “As demonstrated by the numerous prospective clinical studies,” paracetamol is “remarkably safe in therapeutic doses,” without citation	NA: Practice guidelines	5
11. 1972: Double-blind study with two treatments, one each of aspirin and paracetamol, given for antipyretic effect [34]	39 children treated with paracetamol, age 6 months to 6 years	Unspecified “complications or side effects.”	6 h	5
12. 1977: Clinical guidelines for use, predominantly focused on aspirin, but also including paracetamol. [33]	NA: Clinical guidelines	Considered to have a “wide range of safety” based on “the large doses of paracetamol required to evoke toxic reactions” in laboratory animals. In addition, considered “safe and effective when used as directed,” with two studies cited [34, 35]	NA: Clinical guidelines	4
13. 1978: Commentary on paracetamol use [52]	NA: Commentary	Claims: “safe and effective analgesic and antipyretic in usual therapeutic dosage” without citation	NA: Commentary	4
14. 1978: Review comparing aspirin’s and paracetamol’s antipyretic and analgesic activity [53]	NA: Review	Claims: “the choice of agents for antipyresis in clinical practice has been narrowed to aspirin and paracetamol” without citation	NA: Review	4
15. 1996: Review of paracetamol liver toxicity in children under the age of 6 years [54]	NA: Review	Makes no general safety claim, although extensive references are provided showing that paracetamol does not cause long-term damages to infants’ livers	NA: Review	4
16. 1997: Double-blind clinical trial with three treatments, one of paracetamol and two different concentrations of ibuprofen given for fever [55]	97 children treated with paracetamol; median age is 29 months	Renal function as indicated by blood urea nitrogen (BUN) and creatinine levels	4 weeks	4
17. 1972: Clinical study with three treatments, one each of aspirin, paracetamol, and a combination of the two given for fever [35]	80 children treated with paracetamol, age 6 months to 5 years	No outcome measures specified	6 h or less	4
18. 2005: Pharmacokinetic study. [56]	NA: Pharmacokinetic study	No safety outcomes reported. No safety claims made	NA: Pharmacokinetic study	3
19. 2011: Randomized open-label study with two dosing regimens of intravenous paracetamol given for analgesic or antipyretic effect. [57] Intravenous paracetamol contains cysteine, an antidote for paracetamol poisoning. The antidote is not present in the oral medication	75 patients total were treated with paracetamol, 3 neonates, 25 infants, 25 children, and 22 adolescents	Changes in liver enzymes, changes in vital signs, and reported or observed adverse drug effects, which included the following: anemia, constipation, nausea, vomiting, face edema, pyrexia, hypokalemia, hypomagnesemia, hypophosphatemia, agitation, atelectasis, pleural effusion, pulmonary edema, stridor, wheezing, periorbital edema, and pruritus	48 h	3
20. 1973: Review describing precautions with paracetamol use [58]	NA: Review	Makes no safety claim with respect to pediatric use	NA: Review	2
21. 1981: Review comparing efficacy of aspirin and paracetamol in fever reduction in children [59]	NA: Review	Claims: a “high degree of safety” at therapeutic doses without citation	NA: Review	2
22. 1992: Pharmacokinetic study in adults aged 21–25 years [60]	NA: Pharmacokinetic study	NA: study in adults	NA: Pharmacokinetic study	2
23. 1993: Review [61]	NA: Review	Claims: “Recent data have supported the relative safety (and efficiency) of paracetamol in newborn infants” without citation	NA: Review	2
24. 1996: Double-blind study with two treatments, one of each ibuprofen and paracetamol, given for fever [62]	47 children were treated with paracetamol, age 0.2 to 9.4 years; median age is 1.6 years	Extensive assessment of adverse events. Claims: “majority of adverse events had a doubtful or no relationship the treatment, and most were considered mild.”	36 h	2
25. 1997: Computer simulation used to predict dosing needed to achieve desired concentration of drug in plasma [63]	NA: Computer simulation	No safety claims made	NA: Computer simulation	2
26. 1997: Pharmacokinetic study [64]	NA: Pharmacokinetic study	No safety outcomes reported. Claims: “Commonly used in children because of its (efficacy and) safety” without citation	NA: Pharmacokinetic study	2
27. 1999: Pharmacokinetic study with a single rectal dose of paracetamol [65]	28 preterm neonates, 2 days after birth	No safety outcomes stated. Claimed: “safe”	Up to 12 h	2
28. 2007: Comparison of efficacy between paracetamol and ibuprofen. First phase was a single dose, double-blind administered in the clinic, followed by an open-label phase administered at home for the second and subsequent doses. [66]	150 patients treated with paracetamol, age range approximately 0.40 to 11 years; average age is 3.71 years	Monitoring for “adverse events,” three of which were infections, gastrointestinal disorders, and respiratory disorders	3 days	2
29. 2008: Retrospective study using data collected in neonates treated with intravenous paracetamol. [67]	149 neonates total, median postmenstrual age 38 weeks and median postnatal age is 5 days	Hepatic enzyme profiles including ALT, AST, and GGT	48 h	2
30. 2011: Review describing NSAIDs and paracetamol and their roles in reducing side-effects after surgery [68]	NA: Review	Makes no safety claim	NA: Review	2
31. 2013: Review describing dosing and antipyretic efficacy of paracetamol [69]	NA: Review	Claims: “dosing range is well tolerated in children” without citation	NA: Review	2
32. 1965. Pharmacology reference book [70]	NA: Review	Pediatric dose stated without citation, and without further discussion of pediatric use. Makes no safety claim	NA: Review	1
33. 1967: Clinical comparison of a single dose of paracetamol, aspirin, and salicylamide [71]	50 infants treated with paracetamol, up to 48 months old	Unspecified “undesirable effects” not observed	6 h	1
34. 1978: Review describing the pathophysiology of aspirin overdosage [72]	NA: Review	Does not discuss paracetamol. Makes no safety claims	NA: Review	1
35: 1982. Monitoring of drug use by the Pediatric Drug Surveillance Program [73]	1158 children, up to age 16, received paracetamol	Adverse events not reported. Makes no safety claim	NA: No follow-up conducted	1
36. 1982: An abstract [74] cited by Ragg, 1997 [75]	Not determined	Not determined	Not determined	1
37. 1984: Prospective study observing adverse drug reactions in pediatric inpatients. [76]	132 children, age not specified, received an antipyretic or analgesics while hospitalized (paracetamol not mentioned)	No side effects observed. No safety claims made	During inpatient stay: time not specified	1
38. 1989: Editorial comparing ibuprofen and paracetamol [77]	NA: Editorial	Claims: “therapeutic doses of either drug [ibuprofen and paracetamol] cause no discernable adverse effects” without citation	NA: Editorial	1
39. 1989: Review assessing pain in neonates and the approaches to postoperative analgesia [78]	NA: Review	Claims: “Recent data have supported the relative safety and analgesic efficacy of paracetamol in newborn infants” without citation	NA: Review	1
40. 1990: Pharmacology reference book [79]	NA: Review	Claims: “usually well tolerated” without citation, but use in pediatric populations is not discussed	NA: Review	1
41. 1991: Review describing paracetamol hepatotoxicity and poisoning in children [80]	NA: Review	Makes no safety claim	NA: Review	1
42. 1992: Review describing the hepatotoxicity of non-steroidal anti-inflammatory drugs [81]	NA: Review	Claims: paracetamol is “normally very safe when used properly” although this statement does not necessarily refer to pediatric use	NA: Review	1
43. 1992: Randomized, double-blind, multidose, parallel-group, variable duration clinical trial with three different concentrations of ibuprofen and one of paracetamol given for pediatric fever [82]	16 children treated with paracetamol; average age is 5.2 years	Adverse events included headache, gastrointestinal effects, sweating, hypothermia, abdominal pain, agitation, nervousness, and adverse experiences related to the respiratory system	24 to 48 h	1
44. 1994: Textbook [83] cited by Wilson, 1995. [84]	NA: Textbook	Not determined	NA: Textbook	1
45. 1996: Double-blind study with two treatments, paracetamol and placebo given for postoperative pain [85]	100 children were treated with paracetamol, age 3 to 14 years	Liver enzymes determined by blood samples	24 h	1
46. 1996: Randomized study with two treatment groups, one of which received paracetamol preoperatively and the other postoperatively [86]	28 children, age 2 to 8 years	Outcome measures included pain scores and the need for rescue analgesics	240 min	1
47. 1997: Double-blind, multicenter study with two treatments, one each of ibuprofen and paracetamol, given for fever [87]	56 children treated with paracetamol, age 8 months to 11 years; average age is 4.2 years	Changes in temperature. Only side effect reported was vomiting	6 h	1
48. 1999: Randomized, double-blind, placebo-controlled study with four different concentrations of paracetamol given after induction of anesthesia [88]	90 children treated with paracetamol, age 1 to 7 years	Postoperative pain was evaluated by behavioral assessment and physiologic measurement. Only side effects reported were postoperative nausea and vomiting	24 h	1
49. 1999: Clinical trial examining the efficacy and pharmacokinetics of paracetamol in term infants (multiple-dose) [89]	10 infants, up to the age of 2 days	Adverse events not reported. Claims: “paracetamol can be administered safely to neonates on the first day of life.”	First 2 days after birth	1
50. 2000: Review describing non-opioid drugs for treatment of postoperative pain [90]	NA: Review	Claims: rectal paracetamol “seems safe in children” without citation	NA: Review	1
51. 2000: Pharmacokinetic study of postoperative, repeated dosing of rectal paracetamol [91]	21 children, age 9 weeks to 11 years	No sign of adverse effects observed. Claims: “paracetamol has gained wide acceptance as a simple and safe antipyretic and analgesic in children,” without citation	Variable, from 1 to 5 days	1
52. 2000: Observational study of calls to a poison center to evaluate pediatric paracetamol exposures [92]	1019 children up to the age of 7 years	Parent’s report of signs of hepatotoxicity	72 h	1
53. 2000: Review of paracetamol’s history, present, and future [93]	NA: Review	Claims: paracetamol is an “effective and remarkably safe drug when used properly” without citation	NA: Review	1
54. 2000: Randomized, double-blind study with two treatments, one each of diclofenac and paracetamol for postoperative analgesia [94]	24 children treated with paracetamol, age 5 to 15 years; median age is 10 years	Outcome measures used were pain scores and relief of pain or dysphagia. Only side effects reported were nausea and vomiting	3 days	1
55. 2000: Integrated Management of Childhood Illness handbook by the World Health Organization [95]	NA: Review	Makes no safety claims	NA: Review	1
56. 2000: Randomized, double-blind, multicenter study comparing paracetamol controlled-release sprinkles and paracetamol immediate-release elixir in febrile children [96]	120 patients, age 2 to 11 years	Disorientation, extreme irritability, and confusion were the only adverse events recorded Claims: “Both APAP formulations were well tolerated.”	8 to 10 h	1
57. 2000: Guide to pediatric medication and nutrition [97]	NA: Review	Claims: “Usually well tolerated when used as directed,” without citation	NA: Review	1
58. 2001: Review describing treatment with paracetamol in infants [98]	NA: Review	Claims: “generally considered a safe drug” without citation but warns of potential toxicity with glutathione depletion	NA: Review	1
59. 2001: Literature review describing perioperative use of high-dose of rectal paracetamol [99]	NA: Review	Claims: “administration of high-dose rectal paracetamol in the perioperative period appears to be safe” without citation	NA: Review	1
60. 2001: Review describing paracetamol toxicity in children [100]	NA: Review	Claims: safety based on NAPQI production and glutathione levels without citation	NA: Review	1
61. 2001: Review describing the neurobiology of pain [101]	NA: Review	Makes no safety claim	NA: Review	1
62. 2001: Randomized, stratified, placebo-controlled, single-dose, double-blind, triple-dummy, single-center, parallel-group study with four treatments, one each of ibuprofen, ketoprofen, paracetamol, and placebo given for postoperative dental pain [102]	NA: minimum age 16 years, average age is 22.2 years	NA: minimum age 16 years, average age 22.2 years	NA: minimum age 16 years, average age 22.2 years	1
63. 2001: Blinded study conducted to observe the analgesic efficacy of rectal and oral paracetamol in two separate groups in children after craniofacial surgery [103]	40 patients, average age is 10.3 years	Paracetamol plasma concentrations and pain scores. Only side effect reported was vomiting. Makes no safety claims	24 h	1
64. 2002: Review comparing the effects of paracetamol, NSAIDs, or their combination in postoperative pain management [104]	NA: Review	Claims: “low incidence of adverse effects” without citation	NA: Review	1
65. 2002: Literature review describing paracetamol and ibuprofen use for fever treatment in children [105]	NA: Review	Claims: “Both drugs appeared well tolerated and no evidence of difference in short-term adverse effects was observed” without citation	NA: Review	1
66. 2003: Editorial describing use of antipyretics [106]	NA: Editorial	Claims: paracetamol is “traditionally considered to be safe based on (a) large clinical experience over (a) long time” without citation	NA: Editorial	1
67. 2003: Erroneous or out of print citation*** [107]	NA: Erroneous or out of print citation	NA: erroneous or out of print citation	NA: Erroneous or out of print citation	1
68. 2003: Review describing anti-inflammatory agents and paracetamol in neonates [108]	NA: Review	Claims: “paracetamol remains the drug of choice for antipyresis in neonates” and “the adverse effect of paracetamol is more favorable” without citation	NA: Review	1
69. 2003: Randomized, double-blind, placebo-controlled study with four treatments groups, ibuprofen, paracetamol, a combination of the two, and placebo. The purpose was to observe the analgesic efficacy of each treatment [109]	80 children treated with paracetamol, age 1 to 6 years; average age is 2.7 years	Adverse events defined as retching, vomiting, abdominal pain, and dizziness	All children were kept in the PACU for 1.5 h. The parents of the children were asked to record the well-being of their child until 24 h after anesthesia	1
70. 2004: Pharmacokinetic study with a single intravenous dose of propacetamol [110]	30 neonates, 24 h after birth	Liver enzymes determined by blood samples	10 h	1
71. 2004: Systematic review assessing the prevalence of aspirin-induced asthma in adults and children and other issues related to the syndrome [111]	NA: Review	Makes no safety claim with respect to pediatric use	NA: Review	1
72. 2005: Review describing paracetamol’s tolerability profile [112]	NA: Review	Claims: “Paracetamol is a very well tolerated drug at therapeutic doses” without citation, although this statement does not necessarily refer to pediatric use	NA: Review	1
73. 2005: Randomized, double-blind study with three treatments, one each of ibuprofen, paracetamol, and placebo given before surgery [113]	25 children treated with paracetamol, age 3 to 12 years	Agitation in recovery measured using Oucher’s scale	24 h	1
74. 2005: Evaluation of pain management guidelines for tonsillectomy [114]	37 children, age 5–11 years	Evaluation of nausea and vomiting	16 to 20 h	1
75. 2006: Practice guidelines [115]	NA: Practice guidelines	Makes no safety claim	NA: Practice guidelines	1
76. 2006: Practice guideline to assist poison center personnel with management of paracetamol poisoning [116]	NA: Guidelines	Makes no safety claim	NA: Guidelines	1
77–79. 2004–2010: Three textbooks [117–119] cited by Karbasi and colleagues [120]	NA: Textbooks	Not determined	NA: Textbooks	1
80. 2007: Review describing paracetamol safety and hepatotoxicity [121]	NA: Review	Claims: “an excellent overall safety record” with infants and children without citation	NA: Review	1
81. 2007. Open-label, single-sequence, multiple-dose study with intravenous paracetamol in adults [122]	NA: study in adults	NA: study in adults	NA: study in adults	1
82. 2007: Randomized double-blind placebo-controlled study with paracetamol given for fever [123]	103 children treated with paracetamol, age 6 months to 6 years; average age is 26.1 months	Outcome measures included fever clearance time, rate of fall of temperature, percent reduction of temperature, proportion of afebrile children, symptomatic improvement, and clinical and biochemical adverse effects. Claims: “considered to be a safe drug at therapeutic levels.”	6 h	1
83. 2007: Randomized, double-blind, placebo-controlled study with three treatments, one each of naproxen, paracetamol, and placebo before the induction of anethesia [124]	30 children treated with paracetamol, age 1 to 6 years, average age is 1.3 years	Need for postoperative rescue fentanyl and the incidence of postoperative nausea and vomiting	Minimum of 2 h	1
84. 2007: Study with zolmitriptan nasal spray, not paracetamol [125]	NA: study not involving paracetamol	NA: study not involving paracetamol	NA: study not involving paracetamol	1
85. 2007: Guidelines for assessment and initial management of fever in children younger than 5 years [126]	NA: Clinical guidelines	Makes no safety claim	NA: Clinical guidelines	1
86. 2007: Review describing systemic analgesics for children [127]	NA: Review	Claims: “when the maximum daily dose of paracetamol is observed, it is well tolerated” without citation	NA: Review	1
87. 2009: Comparative study with three treatments: paracetamol, ketoprofen, and ibuprofen given for fever [128]	112 children were treated with paracetamol, average age about 4 years old	Children were monitored without observation of drug-related side effects. Makes no safety claim	Up to 48 h	1
88. 2007: Randomized, controlled trial in which patients received either paracetamol or placebo for postoperative pain [129]	29 infants were treated with paracetamol, age 0–2 months	Did not report any adverse events. Measured the efficacy of paracetamol, not safety	48 h	1
89. 2009: Review describing the Italian Pediatric Society guidelines on the management of fever in children [130]	NA: Review	Claims: paracetamol is “generally well tolerated” without citation	NA: Review	1
90. 2009: Review describing drugs of choice for sedation and analgesia in the NICU [131]	NA: Review	Makes no safety claims	NA: Review	1
91. 2009: Review describing the perioperative use of paracetamol [132]	NA: Review	Claims: paracetamol is a “safe, well-tolerated drug with proven efficacy” without citation	NA: Review	1
92. 2009: Systematic review of the clinical safety and tolerability of ibuprofen compared with paracetamol in pediatric pain and fever [133]	NA: Review	Makes no safety claims	NA: Review	1
93. 2009: Online survey of anesthetists and the current prescribing practice of i.v. paracetamol [134]	NA: Survey	Makes no safety claims	NA: Survey	1
94: 2010: Hemodynamic study with intravenous paracetamol in neonates [135]	72 neonates, age 1 to 27 days; average age is 3 days	Assessment of hemodynamics. No safety claims made	6 h	1
95: 2010: Review describing postoperative pain management [136]	NA: Review	Makes no safety claim	NA: Review	1
96: 2010: Meta-analysis of efficacy and safety of ibuprofen and paracetamol in children and adults [137]	NA: Review	Claims similar safety profiles between paracetamol and ibuprofen, but makes no absolute safety claim	NA: Review	1
97: 2011: Study of efficacy and safety in adults [138]	NA: study in adults	NA: study in adults	NA: study in adults	1
98: 2011: Literature review of clinical trials of intravenous paracetamol for postoperative pain [139]	NA: Review	Claims: it “has been well known as a safe and effective” without citation	NA: Review	1
99: 2012: Review of efficacy and pharmacokinetics of paracetamol in pediatric patients [140]	NA: Review	Makes no safety claims	NA: Review	1
100: 2012: Retrospective study using data collected on pediatric surgery patients to identify the status and risk factors of major infections [28]	230 patients, age 0 to 15 years, average age is 4.28 years	Postoperative fever and its etiologies, mortality discharge, and rates of re-open sternotomy reintubation	1 year	1
101. 2013: Case series evaluating the efficacy of intravenous paracetamol in preterm infants with hemodynamically significant patent ductus arteriosus (hsPDA) [141]	10 preterm infants, age 2 to 15 days	Pre- and posttreatment levels of liver enzymes	3 days	1
102. 2013: Mechanistic study in laboratory animals [142]	NA: study in laboratory animals	Makes no safety claim	NA: study in laboratory animals	1
103. 2014: Literature review assessing liver toxicity due to paracetamol in children [143]	NA: Review	Claims: “doses of less than 75 mg/kg/day of paracetamol are safe for children younger than 6 years of age” without citation	NA: Review	1
104. 1984: Prospective study evaluating paracetamol overdose and its treatment in young children [144]	417 children, age 14 days to 5 years	Outcome measures were post-overdose blood work, including markers for liver and kidney functions Claims: it “seems to have a wide margin of safety” and is “likely the safest antipyretic for the young child.”	72 h (duration of treatment with antidote (NAC))	0
105. 1996: Pharmacokinetic study of one single oral dose of paracetamol in children with chronic liver disease [145]	13 children, age 7 months to 12 years	Claims: in children with chronic liver disease, “at least for single doses, there is no cause for concern in the use of paracetamol.”	Up to 36 h	0
106. 1997: Randomized, prospective, double-blind study with two treatments, one each of paracetamol and paracetamol plus codeine, and promethazine for premedication and analgesia for myringotomy [146]	95 children, age 1 to 12 years	Side effects considered included vomiting, pruritus, respiratory depression, and agitation Claims: paracetamol is an effective and safe premedication for minor middle ear surgery	2 h	0
107. 2001: Pharmacokinetic study of paracetamol given to children for tonsillectomy pain [147]	182 children, age 6 to 12 years	Liver enzymes were measured 2 to 3 days after surgery Claims: a dose of “40 mg/kg by mouth preoperatively is a safe and effective treatment for postoperative pain relief for children weighing 20–50 kg.”	2 to 3 days	0
108. 2001: Study evaluating occult acetaminophen hepatotoxicity in hospitalized children receiving acetaminophen [148]	100 children, average age 9.3 ± 5.9 years	Claims: “routine use of acetaminophen at therapeutic doses in ill, hospitalized children and adolescents appears safe”	Up to 72 h	0
109. 2001: Randomized, double-blind multinational trial evaluating the antipyretic effects of dipyrone, ibuprofen, and paracetamol [149]	628 children, age 6 months to 6 years	Most adverse events were of gastrointestinal nature, such as vomiting and diarrhea Claims: all three drugs, including paracetamol, appeared “safe and effective in reducing temperature.”	14 days	0
110. 2003: Randomized, single-blind, parallel, multicenter trial with three treatments given for surgery, one of paracetamol and two of ketoprofen lysine, which were given on a body weight basis [150]	85 children, age 6 to 14 years	Outcome measures included non-specified adverse events, physical findings, and vital signs Claims: “perioperative rectal paracetamol doses of 15–20 mg/kg were effective and safe.”	8 h	0
111. 2004: Randomized, double-blind study in which patients either received paracetamol or ibuprofen to treat uncomplicated typhoid fever [29]	80 children, age 2 to 14 years	Outcome measures based on resolution of clinical symptoms and signs, time to discharge from hospital, fever defervescence, and area under temperature–time curve measured with respect to a baseline of 37 $^\circ$ C Claims: “Both antipyretics appeared to be safe.”	4 to 6 weeks (recurrence of typhoid fever only)	0
112. 2008: Prospective, randomized, double-blind, placebo-controlled study comparing the antipyretic efficacy of paracetamol and paracetamol alternated with ibuprofen [151]	38 children, 6 months to 6 years	Adverse events included diarrhea, flatulence, emesis, decreased appetite, epigastric pain, nausea, headache, and insomnia Claims: “Both regiments were well tolerated.”	6 h	0
113. 2011: Randomized, double-blind, placebo-controlled study with three treatments, one each of paracetamol, ketoprofen lysine salt, and placebo given for pain control in children with pharyngotonsillitis [152]	97 children, 6 to 12 years	Four adverse events were observed. These included bronchitis and rash in the ketoprofen lysine salt group, and diarrhea and cough in the placebo group Claims: “a single oral dose of paracetamol or ketoprofen lysine salt are safe.”	4 days	0
114. 2013: Randomized, non-blinded, parallel-controlled trial studying the efficacy and safety profiles of oral paracetamol and ibuprofen with patent ductus arteriosus [153]	160 infants, gestational age up to 34 weeks and postnatal age up to 14 weeks	Outcome measures were the rates of ductal closure of the two drugs and other adverse events such as hemorrhage, kidney failure, and gastrointestinal problems Claims: “this study clearly showed that a two-course regimen of paracetamol for premature infants is safe and feasible”	Up to 72 h	0
115. 2014: Case series evaluating paracetamol effectiveness, safety, and blood level monitoring during patent ductus arteriosus closure [154]	7 infants, gestational age 26 to 30 weeks	Claims: “paracetamol is an effective and safe therapeutic option for PDA closure.”	24 h	0
116. 2014: Case series evaluating the efficacy of IV paracetamol for the treatment of patent ductus arteriosus [155]	8 preterm neonates, gestational age 24 to 28 weeks	Safety measures included serum concentration of liver enzymes, total and direct bilirubin, creatinine, and urea nitrogen Claims: paracetamol can be considered a “safe therapy for the treatment of patent arteriosus in neonates.”	Time not specified: monitored during therapy only, no follow-up	0
117. 2015: Randomized controlled trial comparing enteral paracetamol and intravenous indomethacin for closure of patent ductus arteriosus [156]	77 preterm neonates, average gestational age in the paracetamol group is 28.5 weeks and 28.9 weeks in the indomethacin group	Primary outcome measure was PDA closure. Secondary outcomes included renal impairment, GI bleed, NEC, sepsis, pulmonary hemorrhage, ROP, IVH and PVL, and O2 requirement Claims: “enteral paracetamol is safe but not superior to intravenous indomethacin.”	7 days	0
118. 2016: Randomized study comparing oral acetaminophen and ibuprofen in premature infants with patent ductus arteriosus [157]	120 infants, average age in the paracetamol group is 2.85 days and 3.42 days in the ibuprofen group	Primary outcome measure was PDA closure on echocardiography. Secondary outcomes included the safety of both drugs and adverse events, such as oliguria, IVH, tendency to bleeding, NEC, and death Claims: “the results clearly show that both drugs are well-tolerated and safe.”	Monitored during 3 days of therapy only, no follow-up	0
119. 2016: Case series evaluating IV paracetamol as a treatment for patent ductus arteriosus [158]	11 neonates, gestational age 23 to 30.3 weeks	No adverse or side effects observed Safety was monitored by collecting data regarding serum concentration of liver enzymes, total and direct bilirubin, creatinine, and urea nitrogen Claims: paracetamol can be considered a “safe therapy for the treatment of PDA in preterm infants.”	Monitored during 3 days of therapy only, no follow-up	0

*This article is cited as “Renn E. The antipyretic use of paracetamol versus ibuprofen in a pediatric care setting. Physical Therapy. 2000; 25:395–397.” This reference does not apparently exist. The volume number corresponding to the year 2000 for the journal Physical Therapy is 80, not 25. We were unable to determine what actual article it may have originally referred to. **The Canadian Pediatric Society paper from 1998 was mis-cited as being from 2000 in one instance. ***This article, cited as Kehlet and Werner (2003) from the journal Drugs, Volume 63, pp 15–22 (Spec no 2), does not exist on the journal’s website for unknown reasons

Several studies emerged as popular citations for the claim that paracetamol use in infants or children is safe when used as directed. Only 19 articles were cited more than twice, and the most popular article [26] was cited a total of 13 times (Table 2) by the 218 articles we identified. However, in some cases, well cited articles did not make original claims of safety, and are therefore not included in Table 2. For example, an article by Perrott and colleagues in 2004 [27] was cited a total of 7 total times by the 218 articles we identified. However, Perrott’s article, being a review, does not make original claims of safety, but rather cites additional articles as the authority for assurance of safety (Fig. 1). Thus, Perrott’s article is not included in Table 2 as an original source for the claim that use of paracetamol is safe for infants and children when used as directed.

**Fig. 2:**
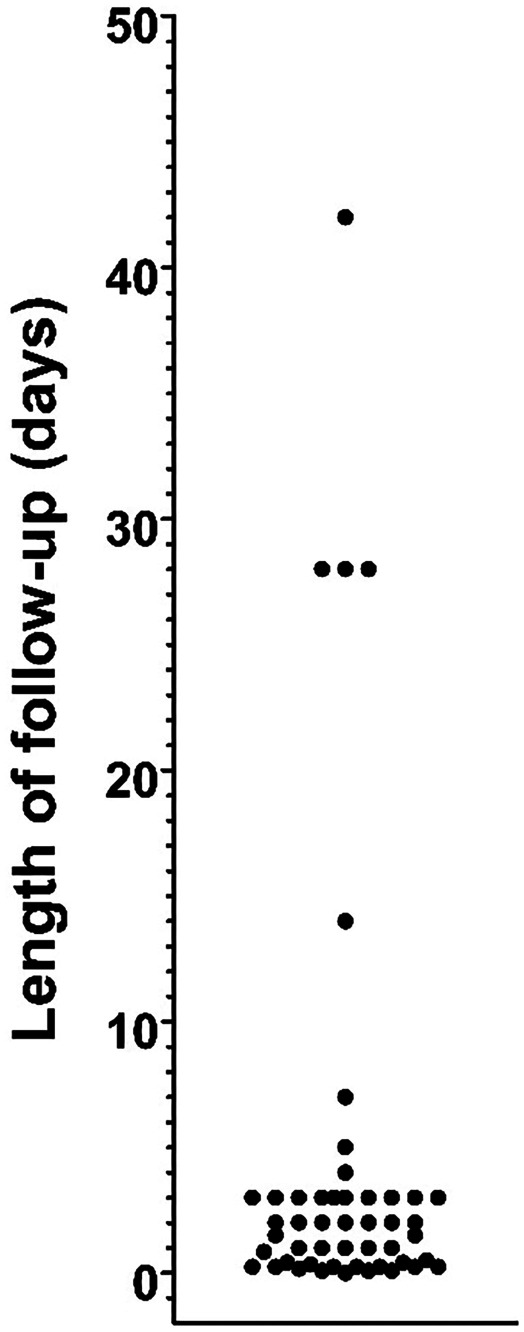
Maximum follow-up times for 49 of the 52 studies describing experiments designed to test the safety of paracetamol in infants or children. One study [28] monitoring readmission for surgery for 1 year is omitted from the graph. Two other studies [76, 155] observing patients during their inpatient visit or treatment period did not specify duration of monitoring, and therefore could not be included in the graph. The five studies monitoring outcomes for 4 weeks or longer did not monitor neuropsychiatric function

Of the 103 articles cited as authority for the safety of paracetamol use in infants or children, 27 did not make claims of safety and did not address safety experimentally (Table 2). Thus, 76 of the 103 articles did address safety, and 48 of these 76 articles (63%) had already been identified in the original 218 articles gleaned from the Medline search. Of the 103 articles, 36 articles described experimental studies which involved paracetamol use in infants or children. As described above, from the original 218 articles making claims of safety, 16 uncited articles described experimental studies that were used to support claims of safety. Thus, 52 studies in total (36 cited plus 16 uncited) provided experimental evidence supporting claims of safety. Although several of those 52 studies provided measures of liver function (Table 2), none of the studies provided any assessment of neuropsychiatric function. Furthermore, the median follow-up time of all 52 studies was 48 h (Fig. 2), far too short to identify any long-term effects of drug exposure on neuropsychiatric function. Six studies had follow-up times of longer than 10 days, although only one study [28] evaluated patients beyond 6 weeks. However, all experimental studies were blind to any potential effects of drug exposure on long-term neuropsychiatric function. For example, although patients were followed for a full year in one study [28], the only endpoint measured was re-admission for surgery. As another example, a study following patients for up to 6 weeks measured only recurrence of typhoid fever beyond the initial treatment period of the study [29].

The path from more recent papers to the original research addressing the safety of paracetamol in infants and children was sometimes convoluted. In one notable case, a popular citation did not did appear in the literature (Table 2). Not only did the volume and journal number not match, but the title could not be found elsewhere. As another example, the citations reporting safety of paracetamol use in children reported by Temple and colleagues in 2017 [30] are illustrated in Fig. 1. This article provides a detailed description of three prior reports to the European Medicines Agency (reports 24,570, 24,571, and 47,402) which, together, according to the authors, “confirm that the recommended standard paracetamol dose of 10 to 15 mg/kg is a safe and effective dose for use in pediatric patients when administered as a single dose or as multiple doses for up to 72 h.” However, the only safety measure used in the three studies was ALT levels as a marker for liver function, assessed for a maximum of 72 h. In addition to the three reports described in their publication, Temple and colleagues cite 10 additional articles as sources for safety, including the claim that paracetamol has a “well-established efficacy and favorable safety profile” (Fig. 1). Among these 10 papers is a clinical trial [31] that addresses efficacy but not safety, and refers to two other papers that address safety, one by Lesko [26]. The paper by Lesko contradicts the view that paracetamol is safe, finding that paracetamol is significantly worse than ibuprofen in terms of risk for outpatient visits following treatment of children with asthma. Another of the articles cited by Temple in 2017, a review written by Temple more than 30 years before [32], cites a paper in the Federal Register [33] as the source for the statement that “Paracetamol is relatively free of side effects and has a wide margin of safety between therapeutic doses and toxic doses.” The document in the Federal Register [33], a lengthy treatise primarily focused on determination of the appropriate dose for adults of salicylates in general and aspirin in particular, in turn cites two papers involving safety studies of paracetamol in the human pediatric population. One of those studies [34] evaluated 98 children using a blinded approach comparing aspirin and paracetamol, and monitored the children for only 6 h. The other study [35] monitored 20 children following administration of both aspirin and paracetamol. In that study, monitoring occurred for 6 h or less, and no information was provided regarding particular side effects that were being assessed. Importantly, the Federal Register [33] attributed their view that paracetamol has “a wide range of safety” to laboratory animal studies showing that the lethal dose of paracetamol is significantly greater than the dose administered to humans. Unfortunately, studies had not been conducted at that time showing that paracetamol induces permanent neurodevelopmental injury in laboratory animals at far lower doses than the lethal dose [19, 20], similar to doses administered to infants and children.

## Discussion

Our initial search of the PubMed^®^ Database and review of more than 3000 titles and abstracts yielded 218 papers making claims that paracetamol is safe for infants and children when used as directed. Claims of safety in those 218 papers were traced back to 103 articles shown in Table 2, but less than 20 of those were cited more than twice, indicating that a limited number of studies are considered key or cornerstone to the view that paracetamol is safe for use in infants or children.

Finding more than 200 articles making claims that paracetamol is safe and/or well tolerated for infants and children when used as directed, this study confirms the view that the drug is widely thought to be safe, despite the absence of any study demonstrating that it is safe for neurodevelopment. The fact that 27 out of 103 articles citated as authority for the safety of the drug did not, in fact, demonstrate safety or make safety claims might suggest that the safety of paracetamol is taken for granted, and is not carefully considered. This view is supported by the observation that one popular citation for safety does not exist in the literature.

This study does not in any way suggest that the effects of early life exposure to paracetamol on neurodevelopment have never been examined. Indeed, the first study to address the issue was published in 2008 by several now-prominent scientists, then at the University of California San Diego and at San Diego State University [22]. This case-controlled, survey-based study raised substantial concerns, as mentioned in the Introduction. Further, studies in animal models evaluating the issue have been conducted [19–21, 36], all indicating that the drug is not safe for neurodevelopment despite a wide range of study designs. In addition, as described in the Introduction, at least 14 cohort analyses [5–18] have indicated that exposure to paracetamol during pregnancy is not safe for neurodevelopment of the fetus. Thus, the present study does not demonstrate that the safety of paracetamol for neurodevelopment has never been examined, but rather demonstrates that assertions that paracetamol is safe during early development when used as recommended are based on a lack of knowledge regarding the effects of paracetamol on neurodevelopment.

The difficulty in moving forward into the clinical arena based on current scientific knowledge is perhaps reflected in the debate surrounding a recent consensus statement supported by almost 100 clinicians and scientists [37] urging caution with the use of paracetamol during pregnancy. This consensus statement of 2021 was met with some skepticism, including an announcement by the American College of Obstetricians and Gynecologists (ACOG) asserting that “Most importantly, patients should not be frightened away from the many benefits of acetaminophen (paracetamol)” [38]. Furthermore, the ACOG asserts that “This consensus statement, and studies that have been conducted in the past, show no clear evidence that proves a direct relationship between the prudent use of acetaminophen (paracetamol) during any trimester and fetal developmental issues” [38]. This latter assertion by the ACOG could technically be considered correct if studies in animal models are ignored, but it demands a level of proof that is not met by the over one dozen studies of cohort data with a wide range of controls for confounding factors via multivariate analysis.

In their response to the 2021 consensus statement, the ACOG clarified their demands for proof, stating that “ACOG’s clinical guidance remains the same and physicians should not change clinical practice until definitive prospective research is done” [38]. However, it is difficult to rationalize the need for such a high level of certainty regarding a drug never demonstrated to be safe or life-saving, where judgment should presumably err on the side of caution and avoidance of harm. Indeed, the drug would not meet current safety standards during preclinical testing due to adverse, long-term neurological effects in laboratory animals, and thus would never reach phase I testing under the current regulatory system. Furthermore, the potential difficulty in obtaining the prospective, controlled study demanded by the ACOG is of concern. Although a study during pregnancy might be envisioned, exposures after birth are likely also important (see Introduction), and therefore must be taken into account in any long-term study. The magnitude and difficulty of a sufficiently powered study, starting from conception and extending into early childhood, is considerable. For example, a group at the University of Oulu conducted a 5-year prospective, placebo-controlled study on 49 children following exposure to paracetamol (*n* = 19) or to saline control (*n* = 20) [39]. However, as the authors point out, their study is underpowered to test the impact of paracetamol on neurodevelopment. In addition, the authors did not control for exposure during all 5 years of the study, but rather only for exposure during a single, 4-day period. Furthermore, it is difficult to imagine a placebo control for treating fevers in babies and children, since withholding paracetamol may need to be accompanied by non-medicinal methods of treating some fevers [40]. Even more importantly, the University of Oulu study used the intravenous formulation of paracetamol rather than the much more commonly used oral formulation. The intravenous formula contains an antidote for paracetamol toxicity (cysteine, a glutathione precursor), which should, hypothetically, block much of the adverse effects of paracetamol. Since this antidote is not present in the commonly used oral formulation, the University of Oulu study, even if it had been much larger and controlled for drug exposure over a period of years, would still not apply to most cases of paracetamol use. It should be noted that, in laboratory animals, exposure during the postpartum period to currently accepted levels of the intravenous formulation of paracetamol with the antidote present causes dramatic increases in asocial behavior later in life [36]. Thus, the argument is not that use of paracetamol with the antidote is safe, but rather that, hypothetically, some of the more serious adverse effects might be prevented by inclusion of the antidote with the drug.

The difficulty in obtaining prospective, controlled studies evaluating the safety of paracetamol in humans is, as outlined above, a complex problem involving large numbers of patients and years of study time, difficulty in establishing controls, and the variable presence of an antidote for paracetamol toxicity in paracetamol formulations. These issues point toward the importance of careful examination of presently available evidence or, as the case may be, the lack of evidence regarding the safety of paracetamol for neurodevelopment.

## Conclusions

Although not the intended purpose of this systematic review with citation tracking, it demonstrated that paracetamol has been proven safe for liver function in infants and in small children, even at doses higher than those currently recommended. During the course of this review with citation tracking, an assumption was repeatedly encountered: because the target of paracetamol toxicity in adults is the liver, demonstration of safety in infants and children need only be tested in the liver. This assumption was/is held despite the fact that the target tissue for drug function is in the central nervous system, not the liver. A similar assumption has proven tragically fatal in the past, when it was assumed that metabolism of the antibiotic chloramphenicol was the same in infants as in adults. In that case, administration of the drug in infants led to a number of deaths [41–43] before the problem was identified.

Despite apparently being taken for granted, this study demonstrates that paracetamol was never shown to be safe for neurodevelopment. This conclusion is consistent with emerging studies showing a connection between paracetamol use during development and long-term neuropsychiatric disfunction as described in the Introduction. This conclusion is also consistent with emerging studies in animal models showing exquisite sensitivity of long-term behavior to early life exposure to paracetamol at near-therapeutic doses.

## Supplementary information

Below is the link to the electronic supplementary material.

Supplementary file1 (DOCX 510 KB)

Supplementary file2 (PDF 152 KB)

## Data Availability

The dataset analyzed (PubMed^®^) is in the public domain.

## Abbreviations

ALT: Alanine aminotransferase
APAP: N-acetyl-para-aminophenol (acetaminophen or paracetamol)
AST: Aspartate aminotransferase
BMI: Body mass index
BUN: Blood urea nitrogen
GGT: Gamma glutamyltransferase
IVH: Intraventricular hemorrhage
NEC: Necrotizing enterocolitis
PDA: Ductus arteriosus
PVL: Periventricular leukomalacia
ROP: Retinopathy of prematurity
