# Supplementary material for: Paracetamol (acetaminophen) use in infants and children was never shown to be safe for neurodevelopment: a systematic review with citation tracking
Source: Eur J Pediatr. 2022 Feb 17;181(5):1835–57. doi: 10.1007/s00431-022-04407-w (PMC9056471; doi:10.1007/s00431-022-04407-w)
Supplement: Supplementary file 1 — Supplementary file1 (DOCX 510 KB) [file 431_2022_4407_MOESM1_ESM.docx]

**Available evidence indicates that pediatric use of acetaminophen adversely affects neurodevelopment in susceptible babies and children.**

**Esha Patel, John P. Jones III, Zacharoula Konsoula and William Parker**

Correspondence: William Parker, Ph.D.

**Running Title**: Acetaminophen and neurodevelopment

**Keywords**: acetaminophen, paracetamol, behavior, neurodevelopment, COX-2, glutathione

**Abstract**

This critical review summarizes the literature pointing toward the safety or lack thereof regarding perinatal use of acetaminophen. Thirteen studies now indicate that prenatal exposure to acetaminophen is associated with neurodevelopmental problems. Based on corrections for confounding factors applied to the analyses of available data, it can be concluded that prenatal exposure to acetaminophen causes statistically significant risks of one subtype of autism spectrum disorder (ASD), developmental delays, and attention deficit hyperactivity disorder. In contrast, data regarding postnatal exposure to acetaminophen are limited, and several factors preclude a classic multivariate analysis of data to resolve the issue. However, circumstantial evidence regarding postnatal exposure to the drug is abundant, and it can be *concluded beyond a reasonable doubt* that postnatal exposure to acetaminophen in susceptible children is responsible for many if not most cases of ASD. Circumstantial evidence includes at least three otherwise unexplained temporal relationships, data from laboratory animal studies, several miscellaneous and otherwise unexplained correlations, and the lack of other suspects.

**Introduction**

Acetaminophen use in babies and in children has been almost universally accepted [1], with exposure to the drug exceeding 90% in some pediatric populations [1-5]. Despite the comfort most health care workers and caregivers have with the drug, a wide range of evidence, reviewed here, indicates that use of acetaminophen in the pediatric population is associated with long term neurodevelopmental problems. A recent, exhaustive review of the literature demonstrates that the safety of acetaminophen use in the pediatric population is an assumption based on numerous studies which show conclusively that the drug does not generally cause liver damage in the pediatric population when used as directed [6]. However, despite the fact that the brain is a primary target organ for the drug’s therapeutic effect, none of the studies making claims of safety ever examined the effect of the drug on neurodevelopment [6]. Further, no study making claims of safety ever considered total acetaminophen exposure since birth, precluding any effective assessment of the drug on neurodevelopment [6]. In the face of widespread but unfounded assumptions of safety, here we will review mounting evidence indicating that use of the drug in the pediatric population carries significant risks for neurodevelopment, and that the effects of the drug might be complex, depending on cofactors associated with oxidative stress and metabolism of the drug.

Based on available evidence, the impact of acetaminophen on the developing brain is much different in the prepartum period compared to the postpartum period. A substantial body of work probing the effects of acetaminophen use during the prepartum has been published, and more than a dozen studies have now established the adverse effects of the drug during the prepartum period. The use of acetaminophen during pregnancy has been associated with a variety of neurodevelopmental problems, including one subtype of autism spectrum disorder (ASD), attention deficit hyperactivity disorder (ADHD), and developmental delays [7-19]. Although the large majority of children suffer no obvious or apparent long-term adverse effects from exposure to acetaminophen, a significant fraction of the population is apparently at risk. The mechanisms by which acetaminophen might profoundly hurt some children while leaving others unharmed have been understood for decades [20-22]: acetaminophen becomes toxic in the presence of oxidative stress (**Figure 1**) due to buildup of a toxic metabolite of acetaminophen, N-acetyl-p-benzoquinone imine. Thus, children with oxidative stress are at increased risk of adverse neurodevelopmental events following exposure to acetaminophen. As shown in Figure 1, a large number of common environmental and genetic factors can cause oxidative stress, and whether a particular child is injured by acetaminophen is determined by both (a) the dose of acetaminophen and (b) the amount of oxidative stress present at the time when exposure to acetaminophen occurs.

In contrast to the numerous studies of the effects of prepartum acetaminophen exposure, studies directly assessing the effects of acetaminophen use during the postpartum period are limited. Nevertheless, considerable evidence regarding the effects of postpartum exposure does exist, and it is possible to piece together a case of sufficient strength to conclude beyond a reasonable doubt that postpartum acetaminophen use is indeed hazardous to neurodevelopment for many susceptible children. In particular, we conclude that postpartum use of acetaminophen is extremely hazardous in babies and children with oxidative stress, and that use of the drug is responsible for many if not most cases of ASD. This conclusion, as will be discussed in this review, is based on a wide range of circumstantial but compelling evidence. With no alternative explanations that can adequately explain the available evidence, the importance of this conclusion cannot be underestimated.

**Prepartum exposure to acetaminophen**

As pointed out in a recent, widely publicized review [23], the long-term effects of acetaminophen use during pregnancy have been the subject of considerable study. A total of 13 studies (Table 1), taken together, demonstrate that acetaminophen use during pregnancy has long-term negative effects on brain function in the offspring. These observations have been affirmed by several meta-analyses [24-26]. Although a negative impact of prenatal acetaminophen use on neurodevelopment is consistently observed, the exact nature of the negative impact covers a wide range. For example, a study in Norway with over 48,000 children revealed that prenatal use of acetaminophen but not ibuprofen had effects on the psychomotor, behavioral, and temperamental development of children at age 3 [9]. Nine out of 13 studies have shown attention-related problems due to acetaminophen use during pregnancy (Table 1). A study in *JAMA Pediatrics* of over 7,000 children, in another example, found prenatal acetaminophen use to be associated with hyperactivity and “emotional symptoms” at age 7 [16], and another study found an association of prenatal use with risk of ADHD at ages 7 and 11 [17]. Additionally, a UCLA study of Danish children found that ADHD is associated with acetaminophen use during pregnancy [19]. Further, prepartum use of the drug is also associated with a subtype of ASD that co-occurs with hyperkinetic disorder [12]. Another study of Danish children found an increased risk of autism-related symptoms and hyperkinetic symptoms associated with prenatal acetaminophen use [14]. Further, a 2017 study found that, while prenatal use of opioids has no association with reduced communication skills, prenatal acetaminophen use did [15]. Thus, based on the preponderance of evidence, it is apparent that exposure to acetaminophen in utero is hazardous for neurodevelopment.

**Studies in laboratory animals**

The industrial revolution has provided humanity with a number of widely used commercial products that are hazardous to neurodevelopment. Such products include phthalates in plastics [27], the pesticide DDT [28], and lead in paint [29]. Studies in laboratory animals have been instrumental in understanding the impact of all of these products on human beings. For example, epidemiological data are still not sufficient to derive quantitative estimates of the hazards of phthalates for human development [30], leaving studies in animal models as a principal guide to creating regulations that limit exposure of humans to these highly toxic compounds [30]. However, laboratory animals may be, at least in some cases, less sensitive to neurodevelopmental toxicity than are humans. For example, the lowest observed adverse effect level of DDT is 50 mg/kg/day in laboratory rats, but only 10.3 mg/kg/day in humans [31]. As another potential example, experimental studies of the neurodevelopmental toxicity of “low levels” of lead in rats typically employ blood lead levels of 0.2 ug/ml or more [32, 33], but blood lead levels of about 0.1 ug/ml and possibly as low as 0.05 ug/ml are dangerous in humans [29].

Studies using both laboratory mice and laboratory rats have shown adverse, long-term effects on neurodevelopment following exposure to acetaminophen shortly after birth (**Table 2**). A Swedish study, for example, found that acetaminophen exposure shortly after birth dramatically impaired the ability of laboratory mice to learn a maze later in life. (**Figure 2A and 2B**) [34]. In that study, mice injected with saline shortly after birth learned rapidly, increasing their speed of running a maze by more than 2-fold after only two days of training. But, based on speed of running the maze, mice injected with acetaminophen shortly after birth lost more than 90% of their ability to learn a maze compared to their saline injected counterparts (p<0.0001). While running the maze, both the control and acetaminophen treated groups had a similar number of errors on day 1 (15.24 in control, 16.95 in treated). However, the control mice averaged less than one error after two days of training, while the acetaminophen-treated mice still averaged more than a dozen errors after the same amount of training (p=0.0003) [34]. These results demonstrate that acetaminophen exposure shortly after birth causes profound, long-term impairment of learning capacity in laboratory mice under the conditions used.

McCarthy’s laboratory has demonstrated long-term neurological changes in laboratory rats following early life exposure to acetaminophen and to other drugs with similar pharmacological activity [35]. A separate study conducted by Duke University researchers on laboratory rats [36], recapitulating conditions of acetaminophen exposure in human infants and children, found that acetaminophen use shortly after birth resulted in an increase in rearing, an asocial behavior, later in life (p<0.0001) (**Figure 2C**). The magnitude of the acetaminophen-induced increase in rearing was concerning: Acetaminophen exposure shortly after birth led to 38% more rearing than in control subjects. With the addition of oxidative stressors such as mock infections and antibiotics, acetaminophen exposure shortly after birth led to 51% more rearing than in control subjects [36].

The significant effect of early life exposure to acetaminophen on learning in mice (Figure 2A and 2B), brain architecture in rats [35], and rearing behavior in rats (Figure 2C) provides convincing evidence of the potential detrimental effects of this drug in human infants and children. However, because many children given acetaminophen are expected to have oxidative stress-inducing conditions such as infection and exposure to antibiotics, and because most studies in laboratory animals use very healthy animals, we expect that some babies and children will be at much greater risk than are laboratory animals. For example, the studies described above on laboratory mice [34] were conducted on healthy animals. Similarly, studies in McCarthy’s laboratory using laboratory rats [35] used healthy animals, and the studies in rats at Duke [36] used antioxidants in conjunction with acetaminophen, reducing the role of oxidative stress in the induction of neurological changes in that model. Further, standard laboratory rodent diets can be so enriched in anti-oxidant vitamins that achieving oxidative stress can be difficult. For example, the commonly used ultra-low fiber, high fat and processed sugar “Western Diet” (Envigo rodent diet TD.88137) used in the Duke study contained 1% weight/volume vitamin mix (Teklad 40060) with a variety of antioxidant B-vitamins. For the sake of comparison, this mass of multivitamin mix is equivalent to more than a dozen multivitamin pills (One-A-Day Women’s 50+: 1.58g/pill) in a typical US diet of 2kg of food per day. In a nutshell, we do not expect previous studies in animals to reflect the potential harm that acetaminophen can cause in susceptible infants and children. To make matters worse, long-held industrial standards in the field of drug development employ screening of laboratory animals only for gross abnormalities in behavior such as seizures, paralysis, and drowsiness [37]. Thus, for example, if a drug caused a profound decrease in socialization or lowered intelligence in a laboratory animal, these adverse effects would not likely be identified using the typical screening methods currently employed by the pharmaceutical industry and approved by the FDA.

Current FDA guidelines dictate that, under experimental conditions, humans should never receive levels of drug above the no observed adverse effects level (NOAEL) in laboratory animals [38]. For example, humans receiving experimental drugs should receive more than 6-fold less drug than levels which cause severe adverse events in laboratory rats, and more than 12-fold less drug than that which causes severe adverse effects in laboratory mice [38]. As discussed above, current levels of exposure to acetaminophen in children cause long-term neurological changes in laboratory animals, which by definition constitute major adverse events. With this in mind, it is apparent that amounts of acetaminophen currently administered to babies and children would not be approved in children using current FDA guidelines for drug approval, and that the levels of the drug currently administered to babies and children exceed by more than 6-fold the dose that should be administered if experimental trials were to be conducted using current guidelines. That being said, the therapeutic dose of acetaminophen covers only a narrow range, and lowering the currently accepted dose of the drug only three-fold renders the drug ineffective [39]. Thus, future experimental clinical trials, if conducted, will necessarily be based on the fact that the drug is already in common use, and will necessarily ignore current regulations for safety.

Exposing babies and children to levels of a drug known to have developmental neurotoxicity in laboratory animals may seem egregious, but the situation is potentially much worse than it appears. As discussed above, laboratory animals previously used for pre-clinical testing of acetaminophen have been very healthy, and it might be expected that unhealthy animals, with oxidative stress, will be much more sensitive to the adverse effects of the drug. Thus, prior work in healthy laboratory animals may dramatically underestimate the dangers of acetaminophen in at-risk babies and children.

**Postpartum exposure to acetaminophen: limited data in human studies**

The first indication that acetaminophen was potentially problematic for neurodevelopment came from Stephen Shultz, who had watched his child regress into ASD following vaccination [40]. In 2008, Shultz and a number of distinguished scientists, then at the University of California San Diego and at San Diego State University, published a small survey study that identified a six-fold increased risk of ASD in one to five year old children when vaccines were accompanied with acetaminophen (odds ratio (OR) = 6.11, 95% confidence interval (CI) 1.42–26.3) but not when the vaccines were given with ibuprofen [4]. The study was immediately criticized for being small and for methodological flaws [41], but a careful analysis of that study reveals a valid design and extremely concerning results [42]. Although the Shultz study was largely ignored for a decade, a meta-analysis of the long-term effects of postpartum acetaminophen exposure was recently published by Alemany and colleagues [24]. The analysis included 6 databases, but only the Danish National Birth Cohort (DNBC) database, with more than 61,000 births, contained data on the occurrence of ASD. Analysis of that databased revealed an OR of 1.30 (95% CI 1.02-1.66) for ASD associated with postnatal paracetamol exposure reported by the mother between birth and 18 months [24]. The analysis of postpartum acetaminophen exposure by Alemany and colleagues was corrected for numerous confounders, similar to most studies of prepartum acetaminophen exposure. Given that levels of postpartum exposure to paracetamol are approaching 100% for at-risk children, the relative risk is expected to be shockingly close to the absolute risk. For example, if all at-risk children are exposed to acetaminophen, then the relative risk of a 30% increase found by Alemany would mean that 23% of all cases of ASD were caused by the exposures to acetaminophen that Alemany considered. (0.77 + 0.23 = 1.0, and 1.0 is 30% more than 0.77.) Equally concerning is the fact that only 7.7% of the mothers providing data for the DNBC database reported giving acetaminophen to their child between birth and 18 months of age. This is an exceptionally low number for children born from 1996-2002, when the children in the DNBC database were born. For example, a study of Danish children born in 2001 found that 65% of the children were exposed to acetaminophen by their mothers *within a three month period* [43]. Thus, it seems possible that the DNBC database underreports acetaminophen administration by mothers between birth and 18 months of age. In addition, Alemany’s meta-analysis [24] did not consider exposure after 18 months, for example during some childhood vaccinations, and it did not consider administration in the hospital, for example during circumcision.

Here it is important to point out that multivariate analyses typically applied to data sets generally try to eliminate the contribution of factors such as antibiotic treatment and infection that are considered to be “confounding”. However, as pointed out above and in Figure 1, these factors are not confounding, per se, but rather are co-factors in the induction of neurological injury. Thus, treating these factors as confounding creates an inherent problem in the analysis. At the same time, the very high prevalence of exposure to the drug [44] creates another inherent problem for multivariate analyses. These problems can come together to literally make the true cause of neurological injury disappear from the analysis. To illustrate the problem, we created an artificial data set (*in silico*) in which 95% of all ASD was caused in simulated (virtual) children by a combination of oxidative stress plus acetaminophen use [44]. To mimic the real world, factors that cause oxidative stress (such as infection and antibiotic use) were associated with acetaminophen use, and the use of acetaminophen reached more than 90% of the population. Despite the fact that ASD was, by definition, caused by exposure to acetaminophen combined with oxidate stress in that population, *only* those factors inducing oxidative stress were identified by the multivariate analysis as being associated with ASD (p < 0.001 for all factors). ASD was not found to be associated with acetaminophen use (p = 0.96) even though acetaminophen had “caused” 95% of the cases of ASD in the simulated data set [44].

Placebo controlled, prospective analysis of children with and without any exposure to acetaminophen followed closely from conception would be, in theory, an ideal way to examine the possibility that acetaminophen use in early development causes ASD [44], but large numbers of children would be required. For example, a study of 1000 children would likely yield 10 to 20 cases of ASD with dozens of potential confounding factors, resulting in a very low-powered study and potentially making it difficult to draw any firm conclusions. Further, such a study would be very expensive and require the better part of a decade to complete. Fortunately, a variety of other evidence is available (**Table 3** and described below) and allows us to conclude that postpartum use of acetaminophen is indeed hazardous to neurodevelopment in children with oxidative stress. Although much of that evidence is indirect, it is sufficient to draw conclusions that are beyond a reasonable doubt. The conclusion that postpartum use of acetaminophen is indeed hazardous to neurodevelopment renders the theoretically ideal study described above unethical and even immoral.

A very small study from the University of Oulu [45] might be considered as evidence that acetaminophen is safe when used in preterm babies. The study follows 19 pre-term patients and 20 pre-term controls given acetaminophen or placebo. The authors of that study do not claim that the drug is safe, but rather end their manuscript with the statement that “*Large randomized trial with standardized follow-up protocol should be conducted to detect any potential association with early neonatal paracetamol treatment and adverse neurological outcomes, like autism spectrum disorders and attention deficit hyperactive disorder.”* However, even if the Oulu study had been larger, that study used the IV acetaminophen preparation, which contains a glutathione precursor that serves as an antidote for excess formation of paracetamol’s toxic N-acetyl-p-benzoquinone imine metabolite. Thus, we do not expect the IV formulation to induce the most severe neurodevelopmental problems. Unfortunately, the much more commonly used oral formulation does not contain the antidote, so children taking the oral formulation are apparently at risk if they have depleted glutathione levels due to extended illness, infection, or genetic/autoimmune issues.

**Postpartum exposure to acetaminophen: overwhelming circumstantial evidence for harm**

The histories of ASD and of acetaminophen use reveal a number of temporal connections. First, the incidence of ASD began to rise rapidly in the early 1980s, coinciding with the early rise in the use of acetaminophen as physicians became aware of the connection between aspirin and Reye syndrome. [45]. Second, as the pediatric community switched from aspirin to acetaminophen, a qualitative shift in ASD, with less infantile ASD compared to regressive ASD, was observed during the early 1980s [46]. This shift indicated that some factor had been introduced into the population capable of inducing ASD in children even after neurodevelopment had progressed for years. Third, the prevalence of ASD rose dramatically through the 1990s as direct to consumer advertising effectively encouraged parents to use formulations of acetaminophen for infants and children [44]. Although the increase in prevalence of ASD over the past 40 years is due in part to increased awareness, improved funding, and other factors, these factors do not explain the timing of the increase or the sustainability of the increase. Further, studies comparing children side-by-side indicate that some environmental factor is indeed inducing ASD. For example, circumcised boys, often exposed to acetaminophen at the time of the circumcision procedure, have a prevalence of ASD that is 50% more than that in uncircumcised boys [47]. This observation points strongly toward a potent environmental factor that induces ASD at the time of circumcision. The impact of this circumcision-associated induction should not be ignored: If 60% of males are circumcised in a population and 75% of cases of ASD occur in males in that population, then it can be readily calculated that a 50% increase in ASD associated with the procedure means that induction at the time of circumcision accounts for more than 17% of all cases of ASD in that population.

*“Once is happenstance. Twice is coincidence. Three times is enemy action”*

*Ian Fleming, in “Goldfinger”*

As shown in Table 3, a number of additional factors are consistent with the view that early childhood exposure to acetaminophen in the presence of oxidative stress can induce ASD. For example, genetic and autoimmune factors associated with ASD have an influence on acetaminophen metabolism [48], and excessive, population-wide exposure to childhood acetaminophen in the Korean population [49] is associated with exceedingly high levels of ASD [50, 51], (See Table 3). Given no evidence that hazards during prepartum exposure disappear at the time of birth, the studies of prenatal exposure, described above, provide additional circumstantial evidence that postpartum exposure to acetaminophen is not benign. Studies in animal models, described above, also provide compelling evidence that prenatal exposure to acetaminophen is toxic for neurodevelopment. The studies in rats by McCarthy [35], in particular, show that the male brain is more sensitive to the drug than the female brain, potentially explaining the preponderance of males with ASD. Further, acetaminophen is known to impair social functioning in human adults [52-54], indicating that the drug does indeed target aspects of brain function known to be altered in ASD. In addition, the induction of ASD by acetaminophen exposure under conditions of oxidative stress provides a plausible and much needed explanation for the fact that many parents of children with ASD have attributed the induction of ASD to vaccination [55, 56], a medical procedure often associated with acetaminophen exposure.

In summary, numerous lines of largely independent evidence point toward the conclusion that a variety of neurodevelopmental problems, especially ASD, can be induced by early life, post-partum exposure to acetaminophen. The tally shown in Table 3 lists 17 lines of such evidence, both direct and circumstantial. Table 3 also lists potential objections to the conclusion that acetaminophen is toxic during neurodevelopment. Importantly, ten of these objections are either unlikely based on experimental results or verifiably false. Eleven more of the objections have no supporting evidence, and exist solely for the purpose of asserting that acetaminophen toxicity in babies and small children might be safe (“post-hoc assertions”, Table 3). Further, if acetaminophen exposure is *not* toxic to neurodevelopment, then a number of observations remain unexplained. The tally shown in Table 3 describes six unknown factors that must be invoked to account for all observations, and eight largely independent observations that must be attributed to coincidence.

**Prepartum versus postpartum exposure to acetaminophen and ASD**

About half of the evidence presented in Table 3 is consistent with the induction of ASD either before or after birth. However, about half of the evidence in Table 3 is consistent *only* with the induction of ASD post-partum, suggesting that many cases of acetaminophen-induced ASD occur postpartum. Further, all studies examining the effects of prepartum acetaminophen use are potentially confounded by the likely possibility that mothers who rely on acetaminophen for personal use will, in turn, rely on the drug for their babies and children. Although accurate calculations of risk are not possible at present, the amount of ASD induced by prenatal exposure to acetaminophen can be very roughly estimated from the numerous studies outlined in Table 1. In particular, Liew and colleagues [14] found roughly a 50% increase (HR = 1.51 95% CI 1.19–1.92) in ASD with hyperkinetic disorder, but that subset of ASD accounted for only 31% of total cases of ASD in that study. Further, about 45% of the women in the study never reported using acetaminophen, consistent with results in other databases [24]. Given that a significant number of women do not use acetaminophen during pregnancy, the absolute risk of acetaminophen-induced adverse events is lower than the relative risk. Thus, the absolute amount of ASD induced by acetaminophen exposure during pregnancy, although apparently significant, may be less than 10 to 15% of the total, and is unlikely to be greater than 20%. This level of induction of ASD is insufficient to account for the majority of the increase in prevalence of the disorder since the 1970s. In contrast, postnatal exposure could readily account for much of the increase in the disorder since the 1970s, potentially accounting for many if not most cases of ASD today.

**Alternative explanations?**

Objections to the view that early life exposure to acetaminophen causes ASD and other neurodevelopmental disorders abound, but none are credible. The easily falsifiable idea that there is no environmental cause of ASD was discussed above. The medical literature states in hundreds of instances that acetaminophen is safe when used as directed in the pediatric population, and even some experts in the field of research on ASD have openly promoted the idea that the drug is safe. However, a systematic review of the literature demonstrates that the drug was never proven safe for neurodevelopment [57], despite the fact that the drug targets the brain. Another objection can be derived from the “biomarkers” of ASD that are present at birth; the presence of biomarkers at birth means that the disorder must have been present at birth, and not induced after birth. However, those biomarkers are long-established markers for inflammation [58, 59], associated with oxidative stress, and thus are biomarkers for susceptibility to acetaminophen-induced injury. Another objection might be that one drug cannot possibly be responsible for very different types of disorders such as ASD and ADHD. However, numerous investigators have pointed out connections between ASD and ADHD. For example, the two conditions are related in terms of diagnostic issues, treatment considerations, and risk factors [60]; symptoms of both tend to co-occur in many individuals [61-63]; and studies in animals models, described above, suggest that effects of exposure to acetaminophen during early development can be complex.

Importantly, objections to the view that early life exposure to acetaminophen induces many if not most cases of ASD are lacking in alternative explanations, and appear to be more attempts to refute particular lines of evidence rather than an attempt to synthesize the data into a coherent model.

**Discussion and Conclusions**

The connection between postnatal acetaminophen use and ASD has escaped notice thus far for reasons that are perhaps obvious. The diagnosis of ASD is often separated in time from the administration of the drug, imposing some difficulty in determining cause and effect. In cases when both cause and effect happen simultaneously, the reason for giving the acetaminophen, for example vaccination or infection, can be blamed. Certainly, the reductionistic focus of the scientific community on molecular and genetic mechanisms has been profoundly helpful in what amounts to a puzzle of almost unimaginable implications for this planet, but without the exercise of backing away from the small pieces to put the puzzle together, the puzzle cannot be solved. In addition, a consensus bias has developed within the medical community, with an underlying assumption based on ignorance that perinatal use of acetaminophen is safe. Further, many researchers studying molecular/genetic mechanisms have a financial conflict of interest with the view that most cases of ASD are induced by acetaminophen exposure early in life. In addition, parents and doctors alike have become so comfortable with the drug that many exposures, both at home and in the hospital, are overdoses [44]. The fact that the drug is present in dozens of different formulations with various names also encourages overexposure.

The conclusion that postpartum use of acetaminophen causes ASD should, if correct, be predictive of experimental outcomes: We predict that use of acetaminophen during early development of male laboratory rats with oxidative stress, particularly rats with low folate levels [64], will induce a severe syndrome characterized by profound impairment of social interactions and reflective of ASD in humans. Given that rats are highly social animals, similar in that regard to humans and a variety of other pack animals, the experiment should yield the predicted results if the conclusion is correct.

The scientific community may disagree on how strong the evidence is. But there should be no disagreement that (a) the evidence is very concerning, (b) physicians and the public should be notified of the current evidence, and (c) the gravity of the issue demands rapid resolution.

**References**

1. Bono-Lunn, D., et al., *Prevalence of Paracetamol Use During Early Development and the Need for Interventional Safety Studies.* Preprints, 2020.

2. Bittker, S.S. and K.R. Bell, *Acetaminophen, antibiotics, ear infection, breastfeeding, vitamin D drops, and autism: an epidemiological study.* Neuropsychiatr Dis Treat, 2018. **14**: p. 1399-1414.

3. Walsh, A., H. Edwards, and J. Fraser, *Over-the-counter medication use for childhood fever: A cross-sectional study of Australian parents.* Journal of Paediatrics and Child Health, 2007. **43**(9): p. 601-606.

4. Schultz, S.T., et al., *Acetaminophen (paracetamol) use, measles-mumps-rubella vaccination, and autistic disorder. The results of a parent survey.* Autism, 2008. **12**(3): p. 293-307.

5. Betz, M.G. and A.F. Grunfeld, *'Fever phobia' in the emergency department: a survey of children's caregivers.* Eur J Emerg Med, 2006. **13**(3): p. 129-33.

6. Cendejas-Hernandez, J., et al., *Paracetamol (Acetaminophen) Use in Infants and Children was Never Shown to be Safe for Neurodevelopment: A Systematic Review.* Preprints, 2020.

7. Alemany, S., et al., *Prenatal and postnatal exposure to acetaminophen in relation to autism spectrum and attention-deficit and hyperactivity symptoms in childhood: Meta-analysis in six European population-based cohorts.* Eur J Epidemiol, 2021.

8. Avella-Garcia, C.B., et al., *Acetaminophen use in pregnancy and neurodevelopment: attention function and autism spectrum symptoms.* Int J Epidemiol, 2016. **45**(6): p. 1987-1996.

9. Brandlistuen, R.E., et al., *Prenatal paracetamol exposure and child neurodevelopment: a sibling-controlled cohort study.* Int J Epidemiol, 2013. **42**(6): p. 1702-13.

10. Ji, Y., et al., *Association of Cord Plasma Biomarkers of In Utero Acetaminophen Exposure With Risk of Attention-Deficit/Hyperactivity Disorder and Autism Spectrum Disorder in Childhood.* JAMA Psychiatry, 2020. **77**(2): p. 180-189.

11. Liew, Z., et al., *Paracetamol use during pregnancy and attention and executive function in offspring at age 5 years.* Int J Epidemiol, 2016. **45**(6): p. 2009-2017.

12. Liew, Z., et al., *Acetaminophen use during pregnancy, behavioral problems, and hyperkinetic disorders.* JAMA Pediatr, 2014. **168**(4): p. 313-20.

13. Liew, Z., et al., *Prenatal Use of Acetaminophen and Child IQ: A Danish Cohort Study.* Epidemiology, 2016. **27**(6): p. 912-8.

14. Liew, Z., et al., *Maternal use of acetaminophen during pregnancy and risk of autism spectrum disorders in childhood: A Danish national birth cohort study.* Autism Res, 2016. **9**(9): p. 951-8.

15. Skovlund, E., et al., *Language competence and communication skills in 3-year-old children after prenatal exposure to analgesic opioids.* Pharmacoepidemiol Drug Saf, 2017. **26**(6): p. 625-634.

16. Stergiakouli, E., A. Thapar, and G. Davey Smith, *Association of Acetaminophen Use During Pregnancy With Behavioral Problems in Childhood: Evidence Against Confounding.* JAMA Pediatr, 2016. **170**(10): p. 964-970.

17. Thompson, J.M., et al., *Associations between acetaminophen use during pregnancy and ADHD symptoms measured at ages 7 and 11 years.* PLoS One, 2014. **9**(9): p. e108210.

18. Vlenterie, R., et al., *Neurodevelopmental problems at 18 months among children exposed to paracetamol in utero: a propensity score matched cohort study.* Int J Epidemiol, 2016. **45**(6): p. 1998-2008.

19. Ystrom, E., et al., *Prenatal Exposure to Acetaminophen and Risk of ADHD.* Pediatrics, 2017. **140**(5).

20. Albano, E., et al., *Mechanisms of N-acetyl-p-benzoquinone imine cytotoxicity.* Mol Pharmacol, 1985. **28**(3): p. 306-11.

21. Mitchell, J.R., et al., *Acetaminophen-induced hepatic necrosis. I. Role of drug metabolism.* J Pharmacol Exp Ther, 1973. **187**(1): p. 185-94.

22. Mitchell, J.R., et al., *Acetaminophen-induced hepatic necrosis. IV. Protective role of glutathione.* J Pharmacol Exp Ther, 1973. **187**(1): p. 211-7.

23. Bauer, A.Z., et al., *Paracetamol use during pregnancy — a call for precautionary action.* Nature Reviews Endocrinology, 2021.

24. Alemany, S., et al., *Prenatal and postnatal exposure to acetaminophen in relation to autism spectrum and attention-deficit and hyperactivity symptoms in childhood: Meta-analysis in six European population-based cohorts.* Eur J Epidemiol, 2021. **36**(10): p. 993-1004.

25. Masarwa, R., et al., *Prenatal Exposure to Acetaminophen and Risk for Attention Deficit Hyperactivity Disorder and Autistic Spectrum Disorder: A Systematic Review, Meta-Analysis, and Meta-Regression Analysis of Cohort Studies.* Am J Epidemiol, 2018. **187**(8): p. 1817-1827.

26. Gou, X., et al., *Association of maternal prenatal acetaminophen use with the risk of attention deficit/hyperactivity disorder in offspring: A meta-analysis.* Aust N Z J Psychiatry, 2019. **53**(3): p. 195-206.

27. Wang, Y. and H. Qian, *Phthalates and Their Impacts on Human Health.* Healthcare (Basel, Switzerland), 2021. **9**(5): p. 603.

28. Ribas-Fitó, N., et al., *In utero exposure to background concentrations of DDT and cognitive functioning among preschoolers.* Am J Epidemiol, 2006. **164**(10): p. 955-62.

29. Abelsohn, A.R. and M. Sanborn, *Lead and children: clinical management for family physicians.* Canadian family physician Medecin de famille canadien, 2010. **56**(6): p. 531-535.

30. Lioy, P.J., et al., *Assessment of phthalates/phthalate alternatives in children’s toys and childcare articles: Review of the report including conclusions and recommendation of the Chronic Hazard Advisory Panel of the Consumer Product Safety Commission.* Journal of Exposure Science & Environmental Epidemiology, 2015. **25**(4): p. 343-353.

31. *Toxicological profile for DDT, DDE, and DDD : draft for public comment*, U.S. Department of Health and Human Services, Editor. 2019, Agency for Toxic Substances and Disease Registry: Atlanta, GA.

32. Zhu, Z.-w., et al., *Study on the neurotoxic effects of low-level lead exposure in rats.* Journal of Zhejiang University. Science. B, 2005. **6**(7): p. 686-692.

33. Tartaglione, A.M., et al., *Sex-Dependent Effects of Developmental Lead Exposure in Wistar Rats: Evidence from Behavioral and Molecular Correlates.* International journal of molecular sciences, 2020. **21**(8): p. 2664.

34. Viberg, H., et al., *Paracetamol (acetaminophen) administration during neonatal brain development affects cognitive function and alters its analgesic and anxiolytic response in adult male mice.* Toxicol Sci, 2014. **138**(1): p. 139-47.

35. Dean, S.L., et al., *Prostaglandin E2 is an endogenous modulator of cerebellar development and complex behavior during a sensitive postnatal period.* Eur J Neurosci, 2012. **35**(8): p. 1218-29.

36. Suda, N., et al., *Therapeutic doses of acetaminophen with co-administration of cysteine and mannitol during early development result in long term behavioral changes in laboratory rats.* PLoS One, 2021. **16**(6): p. e0253543.

37. Irwin, S., *Comprehensive observational assessment: Ia. A systematic, quantitative procedure for assessing the behavioral and physiologic state of the mouse.* Psychopharmacologia, 1968. **13**(3): p. 222-57.

38. FDA, *Guidance for Industry: Estimating the Maximum Safe Starting Dose in Initial Clinical Trials for Therapeutics in Adult Healthy Volunteers*. 2005.

39. Windorfer, A. and C. Vogel, *[Investigations concerning serum concentration and temperature following oral application of a new paracetamol preparation (author's transl)].* Klin Padiatr, 1976. **188**(5): p. 430-4.

40. Schultz, S., *UNDERSTANDING AUTISM: My Quest for Nathan*. 2013: Schultz Publishing LLC. 92.

41. Cox, A.R. and S. McDowell, *A response to the article on the association between paracetamol/acetaminophen: use and autism by Stephen T. Schultz.* Autism, 2009. **13**(1): p. 123-4; author reply 124-5.

42. Schultz, S.T., *Response to the Letter by Cox and McDowell: Association of Paracetamol/Acetaminophen Use and Autism.* Autism, 2009. **13**(1): p. 124-125.

43. Ertmann, R.K., et al., *The majority of sick children receive paracetamol during the winter.* Dan Med J, 2012. **59**(12): p. A4555.

44. Bono-Lunn, D., et al., *Prevalence of Paracetamol Use During Early Development and the Need for Interventional Safety Studies.* Preprints 2020. **2020110224**.

45. Parker, W., et al., *The role of oxidative stress, inflammation and acetaminophen exposure from birth to early childhood in the induction of autism.* Journal of International Medical Research, 2017. **45**(2): p. 407-438.

46. Rimland, B., *The autism increase: research needed on the vaccine connection*, in *Autism Research Review International*. 2000.

47. Frisch, M. and J. Simonsen, *Ritual circumcision and risk of autism spectrum disorder in 0- to 9-year-old boys: national cohort study in Denmark.* J R Soc Med, 2015. **108**(7): p. 266-79.

48. Alberti, A., et al., *Sulphation deficit in "low-functioning" autistic children: a pilot study.* Biol Psychiatry, 1999. **46**(3): p. 420-4.

49. Hall, C. and M. Smith, *Increased cGMP enforcement has gone international: South Korean action against Johnson & Johnson serves as warning.* White Collar Watch, 2013. **June**.

50. Kim, Y.S., et al., *Prevalence of autism spectrum disorders in a total population sample.* Am J Psychiatry, 2011. **168**(9): p. 904-12.

51. Baird, G., *2.64% of South Korean children aged 7 to 12 have autism spectrum disorders.* Evidence Based Mental Health, 2012. **15**(1): p. 11.

52. Dewall, C.N., et al., *Acetaminophen reduces social pain: behavioral and neural evidence.* Psychol Sci, 2010. **21**(7): p. 931-7.

53. Roberts, I.D., I. Krajbich, and B.M. Way, *Acetaminophen influences social and economic trust.* Scientific Reports, 2019. **9**(1): p. 4060.

54. Durso, G.R.O., A. Luttrell, and B.M. Way, *Over-the-Counter Relief From Pains and Pleasures Alike: Acetaminophen Blunts Evaluation Sensitivity to Both Negative and Positive Stimuli.* Psychological science, 2015. **26**(6): p. 750-758.

55. Freed, G.L., et al., *Parental vaccine safety concerns in 2009.* Pediatrics, 2010. **125**(4): p. 654-9.

56. Bazzano, A., et al., *Vaccine-related beliefs and practices of parents of children with autism spectrum disorders.* Am J Intellect Dev Disabil, 2012. **117**(3): p. 233-42.

57. Cendejas-Hernandez, J., et al., *Paracetamol (Acetaminophen) Use in Infants and Children was Never Shown to be Safe for Neurodevelopment: A Systematic Review. .* Preprints, 2020. **2020110173**.

58. Prosperi, M., et al., *Inflammatory Biomarkers are Correlated with Some Forms of Regressive Autism Spectrum Disorder.* Brain sciences, 2019. **9**(12): p. 366.

59. Masi, A., et al., *The Immune System, Cytokines, and Biomarkers in Autism Spectrum Disorder.* Neuroscience bulletin, 2017. **33**(2): p. 194-204.

60. Antshel, K.M. and N. Russo, *Autism Spectrum Disorders and ADHD: Overlapping Phenomenology, Diagnostic Issues, and Treatment Considerations.* Curr Psychiatry Rep, 2019. **21**(5): p. 34.

61. Yerys, B.E., et al., *Attention deficit/hyperactivity disorder symptoms moderate cognition and behavior in children with autism spectrum disorders.* Autism Res, 2009. **2**(6): p. 322-33.

62. Okyar, E. and I. Görker, *Examining the autistic traits in children and adolescents diagnosed with attention-deficit hyperactivity disorder and their parents.* BMC psychiatry, 2020. **20**(1): p. 285-285.

63. Leitner, Y., *The co-occurrence of autism and attention deficit hyperactivity disorder in children - what do we know?* Frontiers in human neuroscience, 2014. **8**: p. 268-268.

64. Frye, R.E., et al., *Cerebral folate receptor autoantibodies in autism spectrum disorder.* Molecular psychiatry, 2013. **18**(3): p. 369-381.

65. Philippot, G., et al., *Adult neurobehavioral alterations in male and female mice following developmental exposure to paracetamol (acetaminophen): characterization of a critical period.* J Appl Toxicol, 2017. **37**(10): p. 1174-1181.

66. Hoang, V.M., et al., *Prevalence of autism spectrum disorders and their relation to selected socio-demographic factors among children aged 18–30 months in northern Vietnam, 2017.* International Journal of Mental Health Systems, 2019. **13**(1): p. 29.

67. McCarthy, M.M. and C.L. Wright, *Convergence of Sex Differences and the Neuroimmune System in Autism Spectrum Disorder.* Biol Psychiatry, 2017. **81**(5): p. 402-410.

68. Hutabarat, R.M., et al., *Disposition of drugs in cystic fibrosis. III. Acetaminophen.* Clin Pharmacol Ther, 1991. **50**(6): p. 695-701.

69. Kearns, G.L., *Hepatic drug metabolism in cystic fibrosis: recent developments and future directions.* Ann Pharmacother, 1993. **27**(1): p. 74-9.

70. Schultz, S.T., et al., *Acetaminophen (paracetamol) use, measles-mumps-rubella vaccination, and autistic disorder: the results of a parent survey.* Autism, 2008. **12**(3): p. 293-307.

71. Randles, D., et al., *Acetaminophen attenuates error evaluation in cortex.* Social Cognitive and Affective Neuroscience, 2016. **11**(6): p. 899-906.

72. Raz, R., et al., *Differences in autism spectrum disorders incidence by sub-populations in Israel 1992-2009: a total population study.* Journal of autism and developmental disorders, 2015. **45**(4): p. 1062-1069.

73. Levaot, Y., et al., *Autism Prevalence and Severity in Bedouin-Arab and Jewish Communities in Southern Israel.* Community Ment Health J, 2019. **55**(1): p. 156-160.

74. Guerri, C. and S. Grisolía, *Changes in glutathione in acute and chronic alcohol intoxication.* Pharmacol Biochem Behav, 1980. **13 Suppl 1**: p. 53-61.

75. Du, K., A. Farhood, and H. Jaeschke, *Mitochondria-targeted antioxidant Mito-Tempo protects against acetaminophen hepatotoxicity.* Arch Toxicol, 2017. **91**(2): p. 761-773.


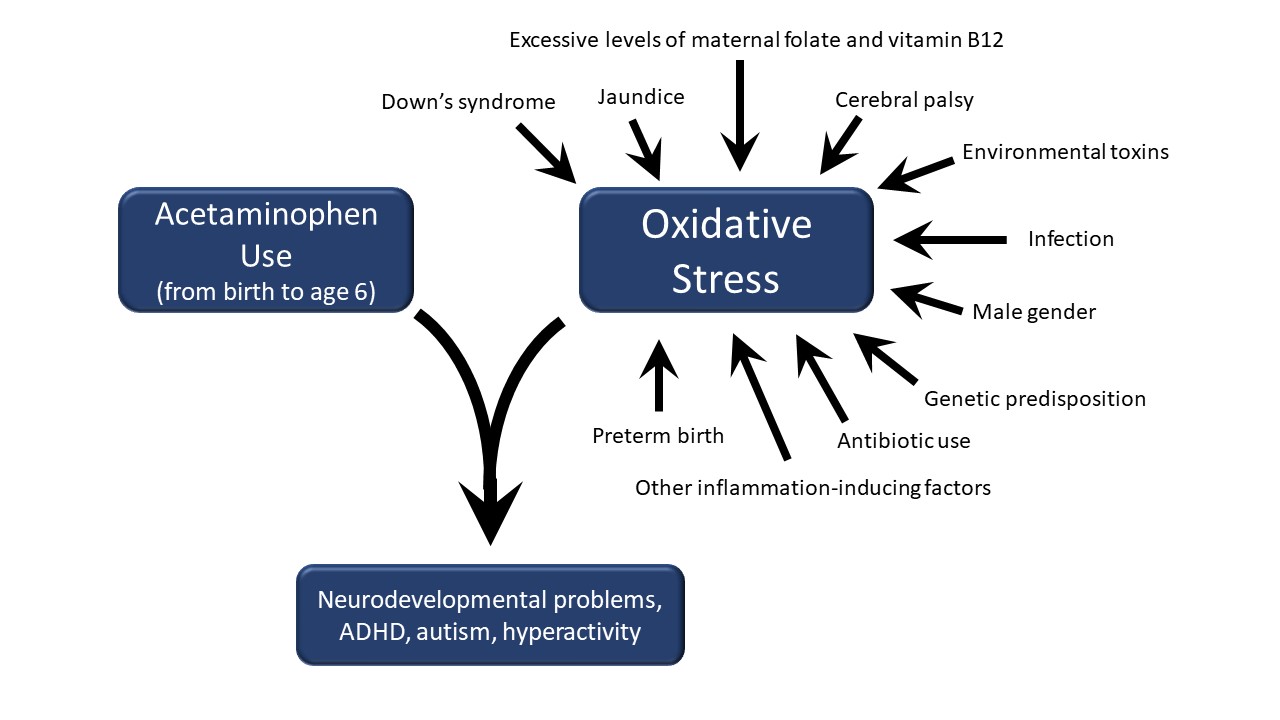


**Figure 1. Effects of early life exposure to acetaminophen may act independently of oxidative stress, but are typically mediated by oxidative stress.**


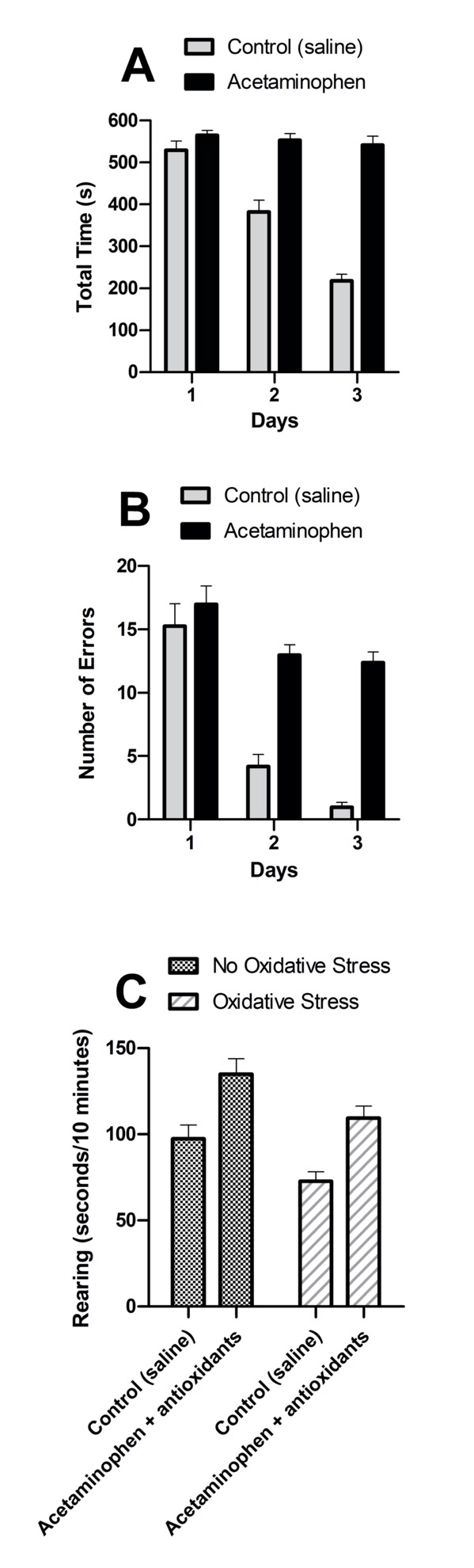


**Figure 2. Long-term effects of early life exposure to acetaminophen on laboratory mice (A and B) and laboratory rats (C).**

Laboratory mice were exposed to either 0.9% saline or two doses of 30 mg acetaminophen/kg bw (administered 4 hours apart) on postnatal day 10, and performance was measured at 2 months of age. After two days of training, mice injected with saline but not mice injected with acetaminophen learned to run the maze (A) faster (p<0.0001) and (B) with less errors (p=0.0003). The rats were exposed to either saline or acetaminophen and antioxidants (cysteine and mannitol) on postnatal days 4 through 10, and rearing behavior (C) was observed on postnatal days 37 through 49. Acetaminophen and antioxidant treatment had 38.4% more rearing behavior than in control rats in an environment without oxidative stress, and 50.6% more in an environment with oxidative stress (p<0.0001). All statistics were obtained through two-way ANOVA tests using GraphPad Prism 9.

**Table 1:** Published studies of the effects of prenatal acetaminophen use on neurological function. [7-19]

| **Study Group** | **Confounding Factors Considered** | **Results (symptoms for children with prenatal acetaminophen exposure)** |
| --- | --- | --- |
| **1**. 48,631 Norwegian children, aged 3 years (compared with ibuprofen use) [9] | Maternal demographics, health (infections, fevers, pain), use of other medications, alcohol use, tobacco use, psychological stress | Problems in psychomotor, behavioral, and temperamental development |
| **2**. 7,796 British children, aged 7 years (compared with maternal postnatal and partner acetaminophen use) [16] | Maternal demographics, health (infections, fevers, pain), genetic risk factors, alcohol use, tobacco use, psychological stress, child’s birth weight, gestational age | Hyperactivity symptoms, conduct problems, “emotional symptoms” |
| **3**. 871 New Zealand children, aged 7 and 11 years (compared with use of anti-inflammatories, aspirin-based painkillers, antacids, and antibiotics) [17] | Maternal demographics, health (fever and inflammation), alcohol use, psychological stress, and medications for psychological conditions | ADHD |
| **4**. 112,973 Norwegian children, aged up to 13 years (compared with maternal acetaminophen use before pregnancy) [19] | Maternal demographics, parental symptoms of ADHD, alcohol use, tobacco use, psychological conditions | ADHD |
| **5**. 64,322 Danish children, aged up to 15.6 years (compared with children not prenatally exposed to acetaminophen) [14] | Maternal demographics, health (infections, fevers, joint and muscle diseases), use of other medications, alcohol use, tobacco use, psychological stress, child’s age and sex | Autism with hyperkinetic symptoms |
| **6**. 64,322 Danish children, aged 7 years (compared with children not prenatally exposed to acetaminophen) [12] | Maternal demographics, health (infections, fevers, pain), alcohol use, tobacco use, psychological stress, child’s birth weight and sex | Hyperkinetic disorders and ADHD-like behavior |
| **7**. 51,679 Norwegian children, aged 3 years (compared with children prenatally exposed to opioids) [15] | Maternal demographics, paternal age and education, tobacco use, psychological conditions, chronic disease, use of other drugs | Reduced communication skills |
| **8**. 73,881 European children, aged 4-12 years (compared with children not prenatally exposed to acetaminophen) [7] | Maternal demographics, health (infections, fevers), alcohol use, tobacco use, mental health problems, child’s health/age/sex | ADHD and borderline or clinical ASD |
| **9**. 2,644 Spanish children, aged 1 and 5 years (compared with children not prenatally exposed to acetaminophen) [8] | Maternal demographics and health (fever, infection, chronic illness), parental mental health, child’s health/age/sex | Autism spectrum symptoms in males, attention-related problems in both genders |
| **10**. 996 U.S. children, aged average of 9.8 years (compared with children not prenatally exposed to acetaminophen) [10] | Maternal demographics, health (fever), use of illicit drugs, alcohol use, tobacco use, early childhood lead levels, child’s sex/birth weight/delivery type | ASD and ADHD |
| **11**. 1,491 Danish children, aged 5 years (compared with children not prenatally exposed to acetaminophen) [11] | Maternal demographics, health (infections, fevers, pain), ibuprofen use, aspirin use, alcohol use, tobacco use, psychological conditions, child’s sex | Subnormal attention and executive function |
| **12**. 1,491 Danish children, aged 5 years (compared with children not prenatally exposed to acetaminophen) [13] | Maternal demographics, maternal IQ, health (infections, fevers, joint and muscle diseases), use of other medications, alcohol use, tobacco use, child’s sex | Lower IQ |
| **13**. 51,200 Norwegian children, aged 18 months (compared with children not prenatally exposed to acetaminophen) [18] | Maternal demographics, health (infections, fevers, pain), use of other medications, alcohol use, tobacco use, psychological stress, prenatal folic acid use | Motor milestone delay and communication problems |

**Table 2:** Studies in animal models show the long-term negative effects of postnatal acetaminophen use.

| **Population or study group** | **Age at time of exposure** | **Post-partum exposure during early development (mg acetaminophen/kg body weight)** | **Effects observed later in life** |
| --- | --- | --- | --- |
| Humans | From conception onward | Unlimited duration of treatment, 14.7 mg/kg bw, every 4-6 hours, no more than 5 doses per day | To be determined |
| Rats in Viberg study [34] | Postnatal day 10 | 1 day of treatment, 30 mg/kg bw, two treatments, 4 hours apart | Decreased learning capability, long-lasting effects on cognitive function, altered adult response to acetaminophen |
| Rats in Suda study [36] | Postnatal days 4-10 | 7 days of treatment, < 14.7 mg/kg bw, every 5 hours | Increased rearing (asocial behavior) when encountering a new rat. |
| Mice in Philippot study [65] | Postnatal days 3, 10, 19 | 1 day of treatment, 30 mg/kg bw, two treatments, 4 hours apart | Negative effects on adult behavior, cognitive function, and habituation capability (long-term effects only observed following exposure on days 3 and 10, not on day 19 after the period of rapid brain growth) |
| Rats in Dean study [35] | Postnatal days 7-13 | 7 days of treatment, 40 mg/kg bw, one dose per day | Long-term modifications to brain development and morphology, decreased social interactions and sensory function in males |

**Table 3:** Overview of current evidence consistent with the view that early life, post-partum exposure to acetaminophen causes long-term neurodevelopmental problems, particularly ASD. Alternative explanations that must be true if acetaminophen is not associated with neurodevelopmental problems are also presented. Factors that point toward the importance of postpartum exposure only (not prepartum exposure) are highlighted in gray. All other factors are consistent with neurodevelopmental toxicity from prepartum or postpartum exposure.

| **Summary of evidence that pediatric use of acetaminophen is dangerous, both direct and circumstantial** | **Alternative explanations for evidence that early life exposure to acetaminophen is hazardous for neurodevelopment** | **Quality of alternative explanation** |
| --- | --- | --- |
| 1. Use of acetaminophen in pregnant women is neurotoxic in their babies, with long-term effects that include lower IQ, increased ASD, and increased ADHD. [7-19] | Although acetaminophen use during pregnancy is proven unsafe, it is safe after birth. | **Post-hoc explanation** with no supporting evidence. **Unlikely**: inconsistent with laboratory animal studies. |
| 2. Laboratory mice and rats develop long-term brain damage with early acetaminophen exposure at doses that are similar to or even less than doses received by human babies and children. [34-36, 65] | Acetaminophen use is unsafe in laboratory animal pups, but, at the same weight-adjusted dose, is safe in human infants. (Laboratory animal pups are more sensitive to toxins than are human babies.) | **Post-hoc explanation** with no supporting evidence. **Unlikely**: inconsistent with studies using other toxins, including the heavy metal lead and the insecticide DDT (see text) |
|  | Based on the presence of biomarkers at the time of birth, ASD is present at the time of birth, and therefore cannot be induced by another factor at a later time. | **Not valid**: The “biomarkers” for ASD are biomarkers for oxidative stress, which affects both the use and metabolism of acetaminophen. |
| 3. The incidence of ASD began to increase in the early 1980s, coinciding with the increase in acetaminophen use after aspirin was associated with Reye’s syndrome. [45] | The concomitant beginning of the rise of pediatric acetaminophen use and ASD was **coincidental**. An as-yet **unknown** factor is responsible for the beginning of the rise in ASD in the early 1980s.  (See also alternative explanations for points 4 and 5.) | **Post-hoc explanation** with no supporting evidence |
| 4. After starting to increase in the early 1980s, the incidence of ASD has steadily increased as direct-to-consumer advertising has driven up use of pediatric acetaminophen. | The concomitant rise of pediatric acetaminophen use and ASD over the past 40 years is **coincidental**. An as-yet **unknown** factor is responsible for the rise in ASD over the past 40 years. | **Post-hoc explanation** with no supporting evidence |
|  | ASD is a neurological variation naturally present in some human populations. The perceived increased incidence of ASD over time is not real, but rather results from factors such as increased awareness, changing diagnostic criteria, and funding-generated incentives for diagnosis. | **Disproven**: Studies showing large differences in cohorts tested at the same time (i.e. boys with and without circumcision [47] and Vietnamese children in urban versus rural areas [66]) show that some environmental factor is causing a profound increase in ASD. |
| 5. The ratio of regressive to infantile ASD rose at the same time as pediatric acetaminophen use rose. [46] | The concomitant shift in acetaminophen use and the time of onset of ASD was **coincidental**. An as-yet **unknown** factor is responsible for the shift in time of onset. | **Post-hoc explanation** with no supporting evidence |
| 6. Male circumcision, often performed using acetaminophen as an analgesic, is associated with a dramatic increase in the risk for early-onset (infantile) ASD. [47] | Circumcision itself or some **unknown** factor associated with the procedure affects the risk for ASD, not acetaminophen use during the procedure. | **Post-hoc explanation** with no supporting evidence **Unlikely**: male circumcision is an ancient procedure with no previously known association with ASD. |
| 7. In laboratory animal studies, acetaminophen affects the developing male brain more than the female brain [35]. ASD also affects males more than females [67]. | The mechanism of acetaminophen-induced injury and the incidence of ASD are independent. The fact that both affect males more than females is **coincidental**. | **Post-hoc explanation** with no supporting evidence |
| 8. Despite the fact that acetaminophen targets the brain, acetaminophen use in babies and children was only proven safe for acute side effects, not for neurodevelopment. [6] | The drug has been commonly used for decades, and therefore must be safe. | **Not valid**: a classic consensus bias |
|  | Physicians are experts in this field and their opinion that acetaminophen is safe for babies and children should be trusted. | **Not valid**: pediatricians are required to practice standard of care and must rely on recommendations by regulatory agencies. Further, it is impossible for physicians to read the millions of research articles published each year covering thousands of medical subjects. |
| 9. Analysis of 61,430 babies in the Danish National Birth Cohort found an OR of 1.3 (CI 1.02-1.66) for ASD associated with postnatal acetaminophen exposure [24]. This result is especially concerning since this study may have dramatically undercounted drug exposure. Further, with exposure to the drug in at-risk children reaching saturation, the relative risk is expected to be close to the absolute risk. (See text.) | The reason for giving the acetaminophen rather than the acetaminophen itself may have caused the ASD, or the ASD and the reason for administering the acetaminophen may have a common cause. | **Not likely:** The analysis was corrected for numerous confounding factors. Further, reasons for giving acetaminophen (fever, pain) have been around much longer than acetaminophen without a history of being associated with ASD. |
|  | Although the study was large, the confidence interval covers a wide range, and no incisive conclusions can be drawn. | The large confidence interval despite the large study size reflects in part the difficulty in conducting retrospective studies to analyze the effects of postnatal acetaminophen exposure. |
| 10. Genetic and immune factors associated with an increased risk of ASD have a detrimental effect on the body’s ability to metabolize acetaminophen. [45, 48] | The genetic and immune factors connected with ASD do not exert their effects via altered acetaminophen metabolism, but rather through other, **unknown** mechanisms. | **Post-hoc explanation** with no supporting evidence |
| 11. Cystic fibrosis is associated with unusually efficient (effective) metabolism of acetaminophen [68, 69], and some evidence suggests that the prevalence of ASD may be very low in patients with cystic fibrosis [45]. | The potentially low prevalence of ASD in individuals with cystic fibrosis has not been confirmed by a systematic study. | It is correct that no systematic study has confirmed a low prevalence of ASD in patients with CF. However, CF is rare and perhaps even unique in that it is associated with increased oxidative stress and has *not* been associated with ASD, despite the fact that mental health in patients with the condition has been studied extensively. |
|  | If confirmed, a low prevalence of ASD in patients with CF may have nothing to do with efficient metabolism of acetaminophen in that population. The association is **coincidental**. | **Post-hoc explanation** with no supporting evidence |
| 12. Acetaminophen given alongside vaccine administration is associated with ASD. [70] | The study was small, and therefore the increased risk observed was possibly due to chance or **coincidence**. | **Post-hoc explanation** with no supporting evidence |
|  | The vaccine itself affects risk for ASD, not the acetaminophen given with the vaccine. | **Disproven**: it has been proven that vaccines alone do not cause ASD. |
| 13. Many parents believe that their children’s ASD was induced by a vaccine based on their own observations or the observations of trusted social networks. [55, 56] | The onset of ASD and the administration of vaccines happen to coincide in some cases. Thus, the parents are mistaking a **coincidence** for a causal event. | **Unlikely**: the observations of parents have proven accurate in the past despite widespread disbelief in the academic community. However, the conclusions of the parents regarding vaccines are incorrect in this case. |
| 14. Acetaminophen use in adults temporarily blunts social trust [53] and awareness [52], emotional responses to external stimuli [54], and the ability to identify errors [71], indicating that the drug targets regions of the brain affected in patients with ASD. | Because the drug blunts social awareness and cognition temporarily in adults does not raise concerns that the drug might cause similar but permanent alterations in babies and small children. The fact that the drug’s effects in adults share similarities with suspected adverse events in children is **coincidental**. | **Post-hoc explanation** with no supporting evidence |
| 15. Ultra-Orthodox Jews [72] and Arabs [72, 73] in Israel have a reported prevalence of ASD less than half of that of other Israelis. Israelis have high rates of circumcision concomitant with ritual use of alcohol. Alcohol use depletes glutathione, particularly in the brain [74], thereby increasing susceptibility to acetaminophen-induced injury. Thus, use of traditional circumcision practices without acetaminophen by some communities in Israel could account in part for their lower rates of ASD compared to other Israelis. | The “mesirah doctrine” practiced by the ultra-Orthodox Jewish community will result in under-reporting of behavior-related pathology. Further, Arab communities in Israel do not access government services efficiently, leading to an artificially low measurement of the prevalence of ASD. | The objections are plausible, although the potential role of acetaminophen plus depletion of glutathione by alcohol during circumcision in the etiology of ASD is unknown. |
| 16. An unexpectedly high prevalence of ASD was identified in South Korea [50, 51], where acetaminophen-containing products for children were repeatedly found to contain amounts of drug exceeding the package label [49]. | Correlations between high rates of ASD and mislabeled acetaminophen products for children in South Korea are coincidental. An **unknown** factor accounts for the high rates of ASD in South Korea. | **Post-hoc explanation** with no supporting evidence |
| 17. Acetaminophen is known to be highly toxic in the presence of oxidative stress. The mechanism by which this toxicity occurs has been established for decades, and involves the formation of the potent toxin, NAPQI [20-22]. More recent studies indicate that concomitant mitochondrial damage [75] is important in the process. | The presence of a plausible mechanism does not prove that the event happens. | The objection is valid. However, the presence of a plausible mechanism provides credibility to the view that acetaminophen is toxic during neurodevelopment. |
